# Traditional Chinese medicine prescription Shenling BaiZhu powder to treat ulcerative colitis: Clinical evidence and potential mechanisms

**DOI:** 10.3389/fphar.2022.978558

**Published:** 2022-09-06

**Authors:** Jing Chen, Bixin Shen, Zhengli Jiang

**Affiliations:** ^1^ Department of Pharmacy, Taizhou Hospital of Zhejiang Province Affiliated to Wenzhou Medical University, Lin Hai, China; ^2^ Department of Pharmaceutics, School of Pharmaceutical Sciences, Wenzhou Medical University, Wenzhou, China

**Keywords:** shenling baizhu powder, ulcerative colitis, clinical evidence, mechanism, signal pathway

## Abstract

Ulcerative colitis (UC), characterized by syndromes including abdominal pain, bloody stool, diarrhea, weight loss, and repeated relapse, is a non-specific inflammatory intestinal disease. In recent years, with the changing dietary habits in China, the incidence of UC has shown an upward trend. UC belongs to the category of recorded as “diarrhea,” “chronic dysentery,” and “hematochezia” in traditional Chinese medicine (TCM), and Shenling BaiZhu powder (SLBZP) is one of the most effective and commonly used prescriptions. In this review, we aim to systematically summarize the clinical application and pharmacological mechanism of SLBZP in the treatment of UC to provide a theoretical basis for its clinical use and experimental evaluation of SLBZP. Our results showed that both SLBZP and SLBZP in combination with chemical drugs, have a significant therapeutic effect against UC with few adverse reactions. Furthermore, combined therapy was better than western medicine. Further, pathophysiological studies indicated that SLBZP has anti-inflammatory, immunomodulatory, antioxidant effects, regulation relative cell signal transduction and regulation of gut microbiota. Although evidence suggests superior therapeutic efficacy of SLBZP for treating UC and the relative mechanism has been studied extensively, various shortcomings limit the existing research on the topic. There is a lack of UC animal models, especially UC with TCM syndromes, with no uniform standard and certain differences between the animal model and clinical syndrome. The dosage, dosage form, and therapeutic time of SLBZP are inconsistent and lack pharmacological verification, and clinical trial data are not detailed or sufficiently rigorous. In addition, SLSZP is composed of multiple Chinese drugs that contain massive numbers of ingredients and which or several components contribute to therapeutic effects. How they work synergistically together remains unknown. Therefore, on the one hand, large sample prospective cohort studies to clarify the clinical efficacy and safety of SLBZP in the treatment of UC are needed. In contrast, researchers should strengthen the study of the molecular biological mechanism of active ingredients and its synergistic actions, clarifying the mechanism of SLBZP in treating UC by multi-component, multi-target, and multi-pathway.

## 1. Introduction

Ulcerative colitis (UC) is a non-specific inflammatory intestinal disease typically characterized by inflammation of the mucosa and submucosa of the rectum and colon (Ungaro et al., 2017). The primary clinical symptoms of UC include abdominal pain, bloody stools, diarrhea, weight loss, and repeated relapse and remission, which seriously affects the quality of life (Ungaro et al., 2017). Currently, the pathogenesis of UC is not adequately clarified and is considered to be associated with the genetic background, environmental factors, and immune dysregulation. In recent years, with changing dietary habits and lifestyle, the incidence of UC has increased in both developed and developing countries, for example in China (Kobayashi et al., 2020). Current treatments of UC mainly include pharmacotherapy and surgical treatment. The therapeutic options include corticosteroids, aminosalicylates, immunosuppressives, such as mesalazine, 5-aminosalicylates, sulfasalazine. Although these drugs could improve symptoms in patients with UC, long-term treatment can lead to vomiting and other adverse effects, resulting in poor compliance. Therefore, there is an urgent need to identify highly efficient drugs with less adverse effects to treat UC.

Traditional Chinese medicine (TCM) as a treatment option is widely used to manage UC. In recent years, numerous studies have confirmed that TCM has been characterized by multi-component, multi-target, and multi-pathway approaches for treating UC and does not carry side effects (Liu et al., 2022a). UC has not been defined in the classics of TCM, and UC falls under the category of “diarrhea,” “chronic dysentery,” and “hematochizia” in TCM(Liu et al., 2021a). With syndrome differentiation, TCM divides UC into seven categories: large intestine damp-*heat* type, *heat* toxin type, spleen deficiency and dampness type, cold-heat complex pattern type, liver depression and spleen deficiency type, spleen and kidney *Yang* deficiency type, and deficiency of both blood and *yin* type (Zhang et al., 2017). The spleen deficiency and dampness type is the common category of UC, and Shenling BaiZhu powder (SLBZP) is one of the effective remedies used for its treatment (Ma et al., 2019).

SLBZP is a TCM compound commonly used in Chinese clinical practice, originally documented in the Song Dynasty “*Taiping Huimin Hejiju Fang*” (Yang, 2018). The formula is composed of *Atractylodes macrocephala* Koidz. (Bai Zhu), *Poria cocos* (Schw.) Wolf (Fu Ling), *Glycyrrhiza uralensis* Fisch. (Gan Cao)*, Platycodon grandiflorum* (Jacq.) A. DC. (Jie Geng)*, Panax ginseng* C.A.Mey. (Ren Shen)*, Amomum villosum* Lour. (Sha Ren)*, Dioscorea opposita* Thunb. (Shan Yao)*, Coix lacryma-jobi* L. var. *mayuen* (Roman.) Stapf (Yi Yi Ren)*, Nelumbo nucifera* Gaertn. (Lian Zi) and *Dolichos lablab* L. (Bai Bian Dou), and stopping diarrhea, SLBZP has long been used to treat digestive system disease in clinical practice. Additionally, SLBZP is also used to treat disease such as chronic obstructive pulmonary disease (Mao et al., 2021a), non-alcoholic fatty liver disease (Zhang et al., 2018), diabetes (Zhou et al., 2019). Recently, numerous clinical and experimental studies have reported the efficacy and mechanism of SLBZP in the treatment of UC(Ma et al., 2019; Chen et al., 2020; Li et al., 2021a; Li et al., 2021b).

However, further in-depth research on SLBZP for the treatment of UC is needed, focusing on the molecular and cellular mechanism of action with more exhaustive pharmacological studies to substantiate the efficacy of this TCM. This review highlights the studies on the pharmacological properties and clinical applications of SLBZP for the treatment of UC. This review may help expand the application of SLBZP to treat UC in clinical practice, and also provide the reference for further study of classical formulas. Moreover, the review of pharmacological effects of single Chinese herb and its active ingredients from SLBZP for UC performed in this study, which may help clarify the effective ingredients and their synergistic actions when formulated in SLBZP.

## 2 Ulcerative colitis and Shenling BaiZhu powder

### 2.1 Understanding of UC in TCM

**FIGURE 1:**
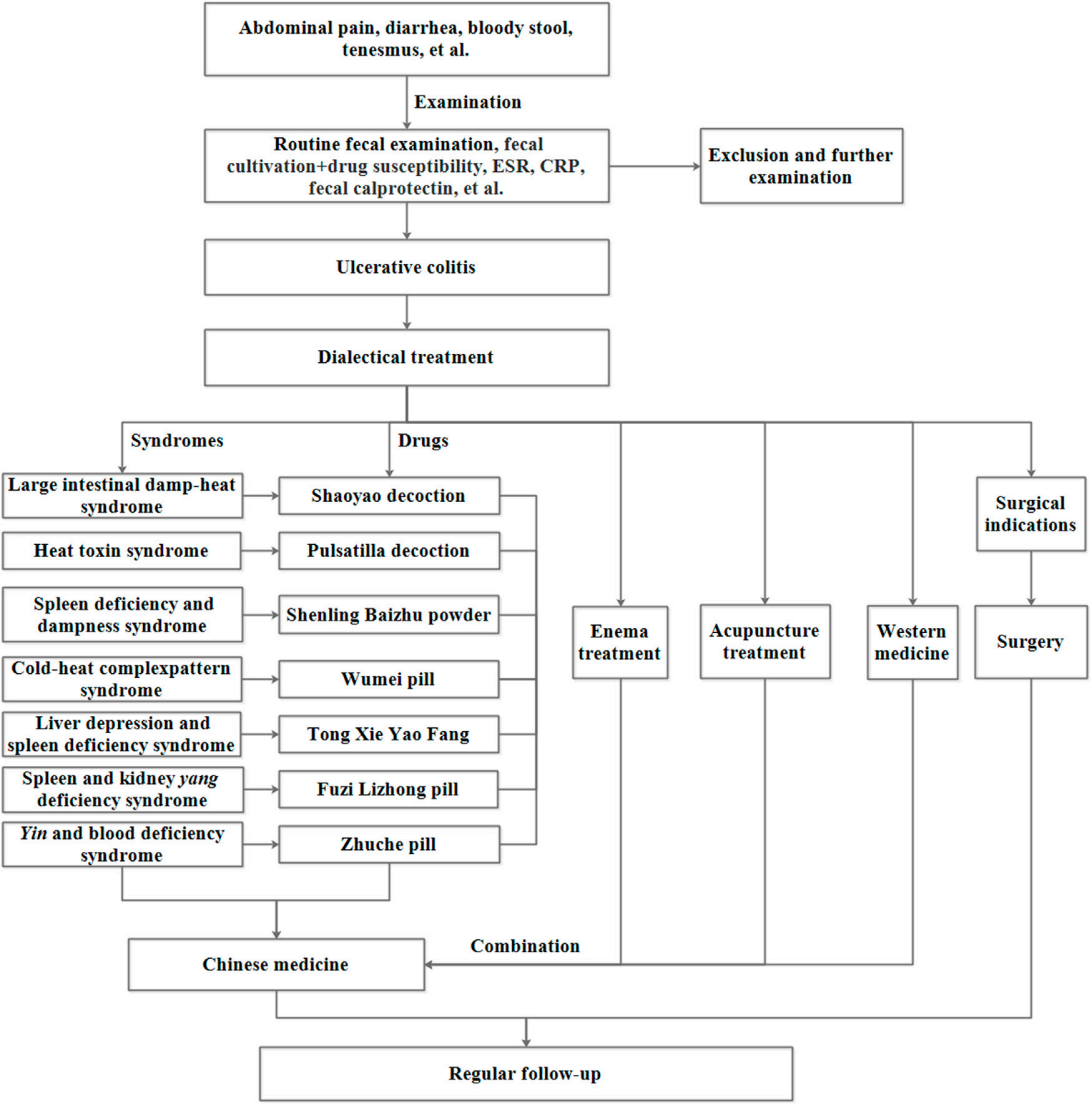
The processes of diagnosis and treatment of UC.

Combining with UC clinical manifestations, including abdominal pain, diarrhea, mucus pus, bloody stool, and tenesmus, UC belongs to the “diarrhea,” “chronic dysentery,” and “hematochezia” category in TCM(Zhang, 2010). Since UC is a chronic disease with recurrence, “chronic dysentery” can describe UC more precisely. Practitioners of TCM believe that the pathogenetic basis of UC is the deficiency of spleen *qi*, and the primary triggers include invasion of external evils, internal injury of emotions, improper diet, and stress (He et al., 2012a). The active phase of UC resembles the TCM excessive syndrome, and the primary pathogenesis is the intestinal accumulation of damp-*heat* and disturbance of *qi* and blood. The pathogenesis of severe UC is *heat*-toxic and stagnant *heat*, and refractory UC with relapse should be blood stasis and turbid phlegm. In remission periods, spleen deficiency is the primary pathogenesis of UC (Liu et al., 2021b). Based on the clinical manifestations and “dialectical treatment” of TCM outcomes, UC is classified into seven subtypes: large intestinal damp-heat syndrome, heat toxin syndrome, spleen deficiency and dampness syndrome, cold-*heat* complicated syndrome, liver depression and spleen deficiency, spleen and kidney *yang* deficiency, and *yin* and blood deficiency syndrome (Zhang et al., 2017). The process for the diagnosis and treatment of UC is shown in Figure 1.

### 2.2 Shenling Baizhu powder

**FIGURE 2:**
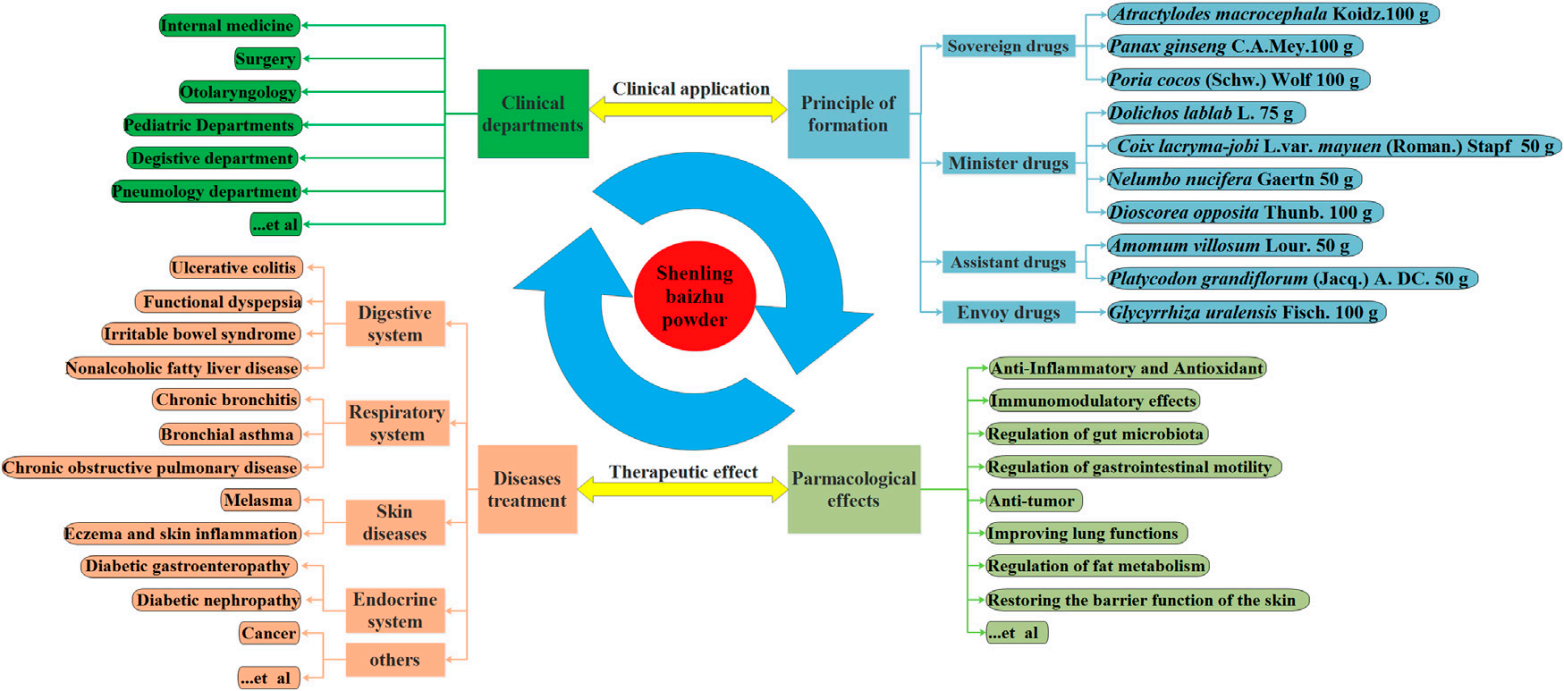
Composition of prescription, clinical applications and therapeutic effects of SLBZP.

SLBZP is composed of 10 Chinese herbal drugs, including *Atractylodes macrocephala* Koidz., *Poria cocos* (Schw.) Wolf, *Glycyrrhiza uralensis* Fisch.*, Platycodon grandiflorum* (Jacq.) A. DC.*, Panax ginseng* C.A.Mey.*, Amomum villosum* Lour.*, Dioscorea opposita* Thunb.*, Coix lacryma-jobi* L. var. *mayuen* (Roman.) Stapf*, Nelumbo nucifera* Gaertn. and *Dolichos lablab* L., which are mainly targeted at spleen deficiency and dampness syndrome. Additionally, SLBZP is a representative prescription of “supplementing spleen to nourish lung” and can be used to treat cough and asthma caused by lung deficiency. The use of SLBZP is widespread in clinical practice and embraces internal medicine, surgery, and postoperative recovery (Lu et al., 2022). Currently, SLBZP is commonly employed to prevent and treat various diseases including UC, irritable bowel syndrome, diabetic gastroenteropathy, diabetic nephropathy, chronic obstructive pulmonary disease, allergic rhinitis, otitis media, non-alcoholic fatty liver disease (Zhang et al., 2018; Ma et al., 2019; Mao et al., 2021b), and in disease mainly involving digestive system, respiratory system, endocrine system, and skin diseases. As showed as in Figure 2.

SLBZP is modified by *Sijunzi* Tang and comes from the “*Taiping Huimin Hejiju Fang*” (Song Dynasty) (Yang, 2018). In this prescription, *Atractylodes macrocephala* Koidz.*, Poria cocos* (Schw.) Wolf*, and Panax ginseng* C.A.Mey., are the sovereign drugs, which can strengthen the spleen and eliminate dampness. As minister drugs, *Dioscorea opposita* Thunb. *and Nelumbo nucifera* Gaertn can help sovereign drugs strengthen the spleen, tonify the *qi*, and relieve diarrhea; *Coix lacryma-jobi* L. var. *mayuen* (Roman.) Stapf and *Dolichos lablab* L. can help sovereign drugs strengthen the spleen and excrete dampness. *Amomum villosum* Lour. and *Platycodon grandiflorum* (Jacq.) A. DC. are the assistant drugs; the former can enliven the spleen and regulate the stomach, promote qi, and relieve dyspepsia, and the latter can diffuse lung and facilitate *qi*, regulate waterways, take herbs bottom-up, and supplement spleen to nourish the lung. *Glycyrrhiza uralensis* Fisch., as the envoy drug, can invigorate the spleen and stomach and coordinate the drug’s actions (Ma et al., 2019). Based on the interactions of these medicines, SLBZP exerts the effects of tonifying the stomach and spleen, eliminating dampness and promoting *qi*, to achieve the aim of treating disease. Modern pharmacological studies have shown that SLBZP has anti-inflammatory (Sun et al., 2020a), antioxidant (Xiong et al., 2021), and immunomodulatory (Li et al., 2014a) effect. It helps regulate gut microbiota (Mao et al., 2021a) and gastrointestinal motility (Lv et al., 2022) and also has anti-tumor (Chen et al., 2018a) potential. Generally, SLBZP is composed of mixed powders of 10 drugs in definite proportions (*Panax ginseng* C.A.Mey.: *Poria cocos* (Schw.) Wolf: *Atractylodes macrocephala* Koidz.: *Dioscorea opposita* Thunb.: *Dolichos lablab* L.: *Nelumbo nucifera* Gaertn: *Coix lacryma-jobi* L. var. *mayuen* (Roman.) Stapf: *Platycodon grandiflorum* (Jacq.) A. DC.: *Glycyrrhiza uralensis* Fisch. = 100: 100: 100: 100: 75: 50: 50: 50: 50: 100) and is to be consumed orally 2–3 times a day at a dose of 3–9 g (Ma et al., 2019).

## 3 Clinical research of SLBZP in treatment of UC

In the clinical prevention and treatment of UC, SLBZP can regulate immune function, decrease pro-inflammatory factors, increase anti-inflammatory factors, and regulate oxidative stress and the balance of gut microbiota (Ma et al., 2019). Additionally, SLBZP can reduce the MMP-2 and MMP-9 levels and ameliorate intestinal mucosal permeability (Wang, 2016). SLBZP, in combination with chemical drugs (such as mesalazine and sulfasalazine) and prescriptions (such as Tong Xie Yao Fang and Sishen pills), is generally prescribed in the current clinical practice. Studies indicated that SLBZP could enhance the therapeutic effects of chemical drugs and reduce side effects and recurrence rates (Xu et al., 2018; Tian et al., 2019). Moreover, powder and decoction are the most commonly used pharmaceutical dosage form of SLBZP, and there are fewer examples of SLBZP pills. Representative clinical studies and its pharmacological data including groups, dosage, treatment time and therapeutic effect are shown in Table 1.

**TABLE 1:** Overview of clinical studies of SLBZP.

Classification	Therapeutic intervention		Treatment time (days)	Evaluation index	Results	References
	Treatment group (UC cases/treatment drugs)	Control group (UC cases/treatment drugs)				
Monotherapy	49 cases; Flavored SLBZP (1 dose per day, decocted in water, thrice daily)	41 cases; Sulfasalazine (oral 2 g, 3 times a day), norfloxacin (oral 0.2 g, 3 times a day) and enema (150 ml, once a day)	30	Clinical efficacy	1) The response in the treatment group was higher than control group	Ren, (2010)
	75 cases; SLBZP (1 dose per day, decocted in water, twice daily)	75 cases; Sulfasalazine (oral 0.5 g, 4 times a day)	60		2) The recurrence rate in the treatment group was lower than control group	Qin, (2012)
	53 cases; Flavored SLBZP (1 dose per day, decocted in water, twice daily)	53 cases; Sulfasalazine (oral 1 g, 4 times a day)	60			Xie, et al. (2015)
	24 cases; SLBZP (according to the prescription powder the herbs and mixed, 6 g each dose, thrice daily)	24 cases; Mesalazine (oral 1 g, 4 times a day)	90	Clinical efficacy, inflammatory factors and gut microbiota	1) The response in the treatment group is higher than control group	He, (2014)
	50 cases; Flavored SLBZP (1 dose per day, decocted in water, twice daily)	50 cases; Mesalazine (oral 1 g, 4 times a day)	168		2) It could decrease the levels of IL-17, TNF-α, IL-23, CRP, ESR and increase the levels of IL-10	Quan and Tan, (2017)
	30 cases; SLBZP (according to the prescription powder the herbs and mixed, 6 g each dose, thrice daily)	30 cases; Mesalazine (oral 1 g, 4 times a day)	84		3) It could promote the growth of probiotics and inhibit the proliferation and colonization of pathogenic bacteria	Yang, (2017)
	26 cases; SLBZP (1 dose per day, decocted in water, twice daily)	26 cases; Mesalazine (oral 1 g, 3 times a day)	30			Yang, et al. (2021)
	84 cases; Flavored SLBZP (1 dose per day, decocted in water, twice daily)	84 cases; Mesalazine (oral 1 g, 3 times a day)	56			Xu, (2021)
	43 cases; Flavored SLBZP (1 dose per day, decocted in water, twice daily)	42 cases; Domperidone (oral 10 mg, 3 times a day)	30	Clinical efficacy	1) The response in the treatment group is higher than control group	Sun and Chen, (2014)
	41 cases; Flavored SLBZP (1 dose per day, decocted in water, twice daily)	41 cases; Compound Sulfamethoxazole and tetracycline	7			Tian and Song, (2015)
	55 cases; Flavored SLBZP (1 dose per day, decocted in water, twice daily)	55 cases; Norfloxacin (oral 0.4 g, 2 times a day)	20			Wang, (2019)
Combination therapy	48 cases; SLBZP (according to the prescription powder the herbs and mixed, 6 g each dose, thrice daily) and Mesalazine (oral 1 g, 4 times a day)	48 cases; Mesalazine (oral 1 g, 4 times a day)	60	Clinical efficacy, flammatory factors, antioxidation, immune function	1) The response in the treatment group is higher than control group	Chen, et al. (2013)
	23 cases; SLBZP (according to the prescription powder the herbs and mixed, 6 g each dose, thrice daily) and Mesalazine (oral 1 g, 4 times a day)	23 cases; Mesalazine (oral 1 g, 4 times a day)	84		2) The response in the treatment group is lower than control group	Wei, et al. (2013)
	42 cases; SLBZP (according to the prescription powder the herbs and mixed, 6 g each dose, thrice daily) and Mesalazine (oral 1 g, 4 times a day)	36 cases; Mesalazine (oral 1 g, 4 times a day)	84		3) It could reduce the levels of IL-17, TNF-α and IL-23, CRP, ESR, IL-1β, IL-6 and IL-18, IL-2, IFN-γ and increase levels of IL-4	Ma, et al. (2015)
	66 cases; SLBZP (granule, 6 g each dose, thrice daily) and Mesalazine (oral 1 g, 3 times a day)	66 cases; Mesalazine (oral 1 g, 3 times a day)	84		4) It could inhibit the expression of NLRP3, ASC and caspase-1 mRNA.	Tian, et al. (2019)
	40 cases; SLBZP (according to the prescription powder the herbs and mixed, 6 g each dose, thrice daily) and Mesalazine (oral 1 g, 4 times a day)	40 cases; Mesalazine (oral 1 g, 4 times a day)	56		5) It could reduce the serum MDA and increase the serum SOD levels	Wang, (2016)
	50 cases; SLBZP (according to the prescription powder the herbs and mixed, 6 g each dose, thrice daily) and Mesalazine (oral 1 g, 4 times a day)	50 cases; Mesalazine (oral 1 g, 4 times a day)	56		6) It could reduce percentage of Th17 cells, IgA, IgM levels and increase the ratio of CD4^+^, CD4^+^/CD8^+^ T cells, the percentage of Treg cells	Zhou, et al. (2018)
	49 cases; Flavored SLBZP (1 dose per day, decocted in water, twice daily)	45 cases; Mesalazine (oral 1 g, 3 times a day)	180		7) It could reduce the MMP-2, MMP-9 levels of UC patients	Shi et al. (2018b)
	31 cases; SLBZP (according to the prescription powder the herbs and mixed, 6 g each dose, thrice daily) and Mesalazine (oral 1 g, 4 times a day)	31 cases; Mesalazine (oral 1 g, 4 times a day)	60			Qian and Zhang, (2019)
	45 cases; Flavored SLBZP (1 dose per day, decocted in water, twice daily)	45 cases; Mesalazine (oral 0.5 g, 3 times a day)	56			Li and Wang, (2021)
	51 cases; SLBZP (1 dose per day, decocted in water, thrice daily) and Sulfasalazine (oral 0.5 g, 3 times a day)	51 cases; Sulfasalazine (oral 0.5 g, 3 times a day)	28	Clinical efficacy, flammatory factors, antioxidation, immune function	1) The response in the treatment group is higher than control group	Yang, (2012)
	47 cases; SLBZP (1 dose per day, decocted in water, twice daily) and Sulfasalazine (oral 0.5 g, 3 times a day)	47 cases; Sulfasalazine (oral 0.5 g, 3 times a day)	28		2) The recurrence rate and incidence of adverse reactions in the treatment group is lower than control group	Ouyang, (2015)
	28 cases; SLBZP (according to the prescription powder the herbs and mixed, 6 g each dose, thrice daily) and Sulfasalazine (enema 3 g/50 ml, one time a day)	28 cases; Sulfasalazine (enema 3 g/50 ml, one time a day)	56		3) It could reduce the levels of TNF-α, IL-1β, IL-2, IL-6, IL-8, TNF-α, INF-γ, HIF-α, IGF-1 and MMP-9 and increase levels of IL-10	Wang, et al. (2016)
	25 cases; Flavored SLBZP (1 dose per day, decocted in water, thrice daily) and Sulfasalazine (oral 1 g, 4 times a day)	25 cases; Sulfasalazine (oral 1 g, 4 times a day)	28		4) It could inhibit the expression of NF-κB p65 and promote the expression of β2AR and β-arrestin2 of intestinal mucosa	Yu, (2017)
	31 cases; Flavored SLBZP (1 dose per day, decocted in water, thrice daily) and Sulfasalazine (oral 1 g, 4 times a day)	31 cases; Sulfasalazine (oral 1 g, 4 times a day)	30		5) It could increase the CD3^+^, CD4^+^, CD8^+^ T cells levels	Zhang, (2017)
	46 cases; SLBZP (1 dose per day, decocted in water, thrice daily) and Sulfasalazine (oral 1 g, 4 times a day)	46 cases; Sulfasalazine (oral 1 g, 4 times a day)	56			Xu, et al. (2018)
	53 cases; SLBZP (granule, 6 g each dose, thrice daily) and Sulfasalazine (oral 1 g, 4 times a day)	53 cases; Sulfasalazine (oral 1 g, 4 times a day)	90			Chen, et al. (2020)
	57 cases; Flavored SLBZP + Tao hua decoction (1 dose per day, decocted in water, thrice daily)	57 cases; Mesalazine (oral 1 g, 4 times a day)	90	Clinical efficacy	1) The response in the treatment group is higher than control group	Jiang, (2019)
	64 cases; Flavored SLBZP + Tong Xie Yao Fang (1 dose per day, decocted in water, thrice daily)	40 cases; Compound Diphenoxylate Tablets (oral 50 mg, 3 times a day) and oryzanol (oral 20 mg, 3 times a day)	42			Zhou, (2009)
	20 cases; SLBZP + Shaoyao Gancao decoction (1 dose per day, decocted in water, thrice daily)	16 cases; Sulfasalazine (oral 1 g, 3 times a day)	28			Li, (2013)
	30 cases; Sishen Pills + SLBZP (1 dose per day, decocted in water, thrice daily) + Mesalazine (oral 1 g, 4 times a day)	30 cases; Mesalazine (oral 1 g, 4 times a day)	56	Clinical efficacy, inflammatory factors and intestinal flora	1) The response in the treatment group is higher than control group (*p* <0.05)	Shen, et al. (2021)
					2) It could increase the numbers of bifidobacterium and *lactobacillus* and reduce the number of escherichia coli and the levels of ET, D-lactate and DAO in serum	
	32 cases; Shaoyao decoction + flavored SLBZP (1 dose per day, decocted in water, thrice daily)	34 cases; Mesalazine (oral 1 g, 4 times a day)	168	Clinical efficacy and recurrence rate	1) The total effects of two groups have no significantly difference	Luo, et al. (2014)
					2) Recurrence rate in treatment group were lower than in control group	
	30 cases; Flavored SLBZP + Gegenqinlian decoction (1 dose per day, decocted in water, thrice daily)	30 cases; Sulfasalazine (oral 1 g, 3 times a day)	90	Clinical efficacy	1)The response in the treatment group is higher than control group	Zhang, et al. (2022a)
	55 cases; SLBZP (1 dose per day, decocted in water, thrice daily) + kangfuxin solution (Enema, 50 ml)	55 cases; Sulfasalazine (oral 0.5g, 3 times a day) + Trimebutine Maleate (oral 0.5g, 3 times a day) +Albumin tannate tablet (oral 0.5g, 3 times a day)	30	Clinical efficacy	The response in the treatment group is higher than control group	Chen, (2012)
	43 cases; Flavored SLBZP (1 dose per day, decocted in water, thrice daily) + enema (100 ml, 2 times a day)	37 cases; enema (100 ml, 2 times a day)	42	Clinical efficacy	1) The response in the treatment group is higher than control group	Tian, (2019)
	59 cases; Flavored SLBZP (1 dose per day, decocted in water, thrice daily) + Mesalazine (oral 1 g, 3 times a day) + acupuncture	59 cases; Mesalazine (oral 1 g, 3 times a day)	84	Clinical efficacy and inflammatory factors	1) The response in the treatment group is higher than control group	Hua, (2022)
	47 cases; Flavored SLBZP (1 dose per day, decocted in water, thrice daily) + Mesalazine (oral 1 g, 3 times a day) + warm acupuncture	46 cases; Mesalazine (oral 1 g, 3 times a day)	28		2) it could decrease the serum 5-HT and SP, numbers of intestinal yeast, serum IL-6 and TNF-α levels and increase the levels of SS and VIP, intestinal bifidobacteriu, *lactobacillus*, and numbers of *peptococcus*	Zhou et al. (2021b)
					3) It exhibited immunomodulatory effects by regulating the levels of Th17, Treg, Th17/Treg, HMGB-1, HIF-1α, IGF-1	

### 3.1 Monotherapy

Clinical research has shown that both SLBZP and flavored SLBZP can significantly alleviate the symptoms in UC patients, including diarrhea, abdominal pain, and hematochezia, without significant adverse reactions. Moreover, SLBZP has fewer adverse reactions and better efficacy than chemical drugs. Studies showed that the combined efficacy of SLBZP was significantly higher than sulfasalazine (Ren, 2010; Qin, 2012; Xie et al., 2015). For example, among 150 patients with UC, 75 cases were given SLBZP (treatment group, 1 dose per day, decocted in water, twice daily), while another 75 were treated with sulfasalazine (control group, sulfasalazine, 0.5, 4 times a day), 1 month is a course of treatment, both groups were given medication for 2 courses before reexamination of colonoscopy. The results showed that the total efficiency in treatment group (96.4%) was better than that in control group (80.4%) (Qin, 2012). In another study, 106 cases with UC randomly divided into treatment group (SLBZP, 1 dose per day, decocted in water, twice daily) and control group (sulfasalazine, 1 g, 4 times a day) for 8 weeks. The total effective rate of treatment in the treatment group was higher than the control group, and the recurrence rate in the treatment group was considerably lower than sulfasalazine (Xie et al., 2015). Meanwhile, researchers also compared the efficacy of SLBZP and mesalazine for use in UC(He, 2014; Quan and Tan, 2017; Yang, 2017; Yang et al., 2021). For instance, 48 cases with UC randomly divided into treatment group (SLBZP, according to the prescription powder the herbs and mixed, 6 g each dose, 3 times a day) and control group (mesalazine, 1 g/time, 4 times a day) for 90 days. The total effective rate of treatment in the treatment group (91.67%) was higher than the control group (70.83%) (He, 2014). In addition, flavored SLBZP (1 dose per day, decocted in water, twice daily) could decrease the levels of IL-17, TNF-α, IL-23, CRP, and ESR and increase the levels of IL-10 in UC patients, and these effects were superior to mesalazine (1 g/time, 4 times a day) (Xu, 2021). Furthermore, flavored SLBZP (1 dose per day, decocted in water, twice daily) could promote the growth of probiotics and inhibit the proliferation and colonization of pathogenic bacteria by improving the intestinal microecological environment and protecting intestinal mucosa (Yang et al., 2021). Similarly, several studies indicated that the therapeutical effects of flavored SLBZP (1 dose per day, decocted in water, twice daily) were better than other chemical medicines, including domperidone (Sun and Chen, 2014), sulfamethoxazole and tetracycline combination (Tian and Song, 2015), and norfloxacin (Wang, 2019). The above research showed that SLBZP was superior to chemotherapeutics in improving the pathological changes and clinical manifestations of UC by reducing the inflammatory factors and regulating the balance of gut mucosa. However, there are some differences in the treatment time and dosage form of SLBZP, which may affect the results of the evaluation of therapeutical effects. Meanwhile, the mechanism of action underlying SLBZP monotherapy needs further exploration.

### 3.2 Combination therapy

#### 3.2.1 Combining with chemical drugs

Combination therapy is the most frequently used treatment modality of SLBZP, and mesalazine and sulfasalazine are the most commonly used chemical medicines combined with SLBZP in treating UC. In a trial, 96 cases of UC patients were randomly divided into control group and treatment group, and control group was treated with mesalazine (oral 1 g, 4 times a day) for 8 weeks, while treatment group was treated with the original treatment plan and combined with SLBZP (according to the prescription powder the herbs and mixed, 6 g each dose, 3 times a day) for 8 weeks. Results showed that the total effective rate of treatment in the treatment group was higher than the control group. While some adverse reactions were associated with SLBZP (powder, oral 6 g each dose, 3 times a day) and mesalazine (oral 1 g, 4 times a day) combination treatment, these were not significant (Chen et al., 2013). Meanwhile, numerous studies have now found that combined SLBZP and mesalazine could decrease the levels of CRP, ESR, IL-17, TNF-α, IL-23, IL-1β, IL-18, IL-2, IL-6, IFN-γ and increase the levels of IL-4 (Wei et al., 2013; Ma et al., 2015; Zhou et al., 2018; Tian et al., 2019; Li et al., 2021c). Meanwhile, SLBZP (powder, oral 6 g each dose, 3 times a day) and mesalazine (oral 1 g, 4 times a day) in combination could inhibit the expression of NLRP3, ASC, and caspase-1 mRNA in UC patients (Zhou et al., 2018). Studies showed that SLBZP (flavored SLBZP, 1 dose per day, decocted in water, twice daily) and mesalazine (oral 1 g, 4 times a day) in combination exerted the antioxidant effects by increasing the serum MDA and serum SOD levels (Shi et al., 2018a). Meanwhile, SLBZP (granule/powder, oral 6 g each dose, 3 times a day) and mesalazine (oral 1 g, 3–4 times a day) combination exert immunomodulatory effect by changing the serum IgA and IgM levels as well as the ratio of Th17, Treg, CD4^+^ T, and CD8^+^ T cells (Qian and Zhang, 2019; Tian et al., 2019). In addition, SLBZP (powder, oral 6 g each dose, 3 times a day) and mesalazine (oral 1 g, 3–4 times a day) in combination could decrease the serum MMP-2 and MMP-9 levels, ameliorate intestinal mucosa permeability, and improve the symptoms of UC patients (Wang, 2016).

Compared with mesalazine, the side effects of sulfasalazine were commonly observed in clinical practice (Ungaro et al., 2017). However, studies indicated that flavored SLBZP and sulfasalazine in combination could significantly decrease the side effects such as nausea, emesis, and inappetence, which were caused by sulfasalazine alone when treating UC. Among 56 patients with UC, 28 cases were given sulfasalazine (control group, 1 g/time, 4 times a day), while another 28 were treated with the original treatment plan and combined with flavored SLBZP (treatment group, flavored SLBZP, 1 dose per day, decocted in water, twice daily) for 30 days. The results showed that the total efficiency in treatment group was better than that in control group, and flavored SLBZP and sulfasalazine in combination could significantly decrease the side effects such as nausea, emesis, and inappetence, which were caused by sulfasalazine alone when treating UC (Zhang, 2017). Meanwhile, the recurrence rate was significantly lower than sulfasalazine alone (Yang, 2012; Ouyang, 2015; Yu, 2017). Further, SLBZP (pill, oral 6 g each dose, 3 times a day) and sulfasalazine (oral 1 g, 3–4 times a day) in combination could significantly decrease the levels of IL-2 and INF-γ, and increase the levels of IL-10 in older adults with UC(Zhao et al., 2017). Studies showed that SLBZP (1 dose per day, decocted in water, thice daily) and sulfasalazine (oral 1 g, 4 times a day) in combination could improve immune function and reduce inflammatory response by downregulating the levels of IL-6, IL-8, CD8^+^ T cells and upregulating the levels of CD3^+^, CD4^+^, CD4^+^/CD8^+^(Xu et al., 2018). In addition, SLBZP (granule, oral 6 g each dose, 3 times a day) and sulfasalazine (oral 1 g, 4 times a day) combination could downregulate the levels of IL-1β, IL-6, IL-8, TNF-α, INF-γ, HIF-α, IGF-1, and MMP-9, the expression of NF-κB p65, β2AR, and β-arrestin 2 protein, in turn, restore the injured intestinal mucosa and achieving therapeutic effects (Chen et al., 2020; Chen and Zhang, 2020).

Furthermore, in combination, SLBZP, lacteol fort, and sulfasalazine could improve the syndromes and promote intestinal mucosal repair, and it has no significant adverse effects (Xiang et al., 2017). Moreover, SLBZP combined with bifid triple viable capsule powder composed of *Bifidobacterium*, *Lactobacillus*, and *Streptococcus faecalis* has also been used to treat UC. Studies indicated that in combination, SLBZP and bifid triple viable capsule powder had good therapeutical effects on UC and were better than sulfasalazine (Zhu, 2012; Ma, 2019).

#### 3.2.2 Combining with TCM prescriptions

Besides using a combination of chemical medicines, some studies have been conducted on the SLBZP combination with other TCM prescriptions for treating UC in the clinic. Studies showed that combining flavored SLBZP with TCM prescriptions such as Taohong Siwu decoction, Tong Xie Yao Fang, and Shaoyao Gancao decoction could improve the syndromes of UC patients and the therapeutic effects were superior to monotherapy with chemical medicines (Zhou, 2009; Li, 2013; Jiang, 2019).

Additionally, the different syndromes of UC patients in TCM, including spleen and kidney *yang* deficiency syndrome, *hot* and dampness syndrome, spleen deficiency and dampness syndrome, and liver depression and spleen deficiency syndrome, were respectively treated by combining SLBZP with Sishen pills (Ma, 2021; Shen et al., 2021), Shaoyao decoction (Luo et al., 2014), Gegenqinlian decoction (Zhang et al., 2022a), and Tong Xie Yao Fang (Zhu and Li, 2012). Meanwhile, researchers showed that SLBZP and Sishen pill in combination could significantly decrease CRP and ESR levels, reduce the number of *Escherichia coli,* decrease the levels of ET, D-lactate, and DAO in serum, and increase the numbers of *Bifidobacterium* and *Lactobacillus*, in turn decreasing the inflammatory response and improving the intestinal flora structure and promoting repair of intestinal mucosal barrier (Ma, 2021; Shen et al., 2021).

#### 3.2.3 Combining with enema and acupuncture

Other than oral delivery of therapeutic agents, some studies have examined the combination of SLBZP with enema and acupuncture for treating UC in clinical practice. Treatments involving enema using the kangfuxin liquid (enema, 200 ml, once a day) combined with SLBZP (1 dose per day, decocted in water, twice daily) and mesalazine (oral 1 g, 4 times a day) showed that the effects of combination therapy were better, and the occurrence of adverse reactions was lower than mesalazine alone (Lai et al., 2014; Mu and Xiao, 2016). Meanwhile, combing SLBZP or other prescription enemas, SLBZP and mesalazine in combination for treating UC was widely used to treat UC, and it was found to be highly effective (Chen, 2014; Tian, 2019; Sun and Guo, 2022). Furthermore, a study found that flavored SLBZP (1 dose per day, decocted in water, twice daily), mesalazine (oral 1 g, 3 times a day), and enema (100 ml, once a day) could improve TCM syndrome score and Mayo score, decrease the levels of IL-2, IL-17, TNF-α, INF-γ, and increase the levels of IL-10 in UC patients (Yi et al., 2021). Additionally, warm acupuncture combined with flavored SLBZP (1 dose per day, decocted in water, twice daily) could decrease the serum 5-HT and SP, numbers of intestinal yeast, serum IL-6 and TNF-α levels, and increase the levels of IL-10, SS, and VIP, intestinal *Bifidobacterium*, *Lactobacillus*, and the number of *Peptococcus*, in turn improving the clinical symptoms that may be related to the correction of abnormal brain-gut axis and antagonistic inflammatory response (Zhou et al., 2021a). Meanwhile, flavored SLBZP (1 dose per day, decocted in water, twice daily) and acupuncture in combination exhibited immunomodulatory effects by regulating the levels of Th17, Treg, Th17/Treg, TNF-α, HMGB-1, HIF-1α, and IGF-1 (Hua, 2022).

### 3.3 Meta-analysis on effectiveness and safety of SLBZP in treatment of UC

Evidence based medicine (EBM) is the dominant paradigm in assessing the effectiveness of clinical treatments. The processes of EBM mainly contain several steps: 1) pose questions; 2) search relative data; 3) quantitative statistical analysis; 4) systematic evaluation; 5) promote the effective treatment methods and abandon ineffective, or even harmful, treatment methods (Djulbegovic and Guyatt, 2017). Systematic reviews, including a quantitative and qualitative evaluation, are currently considered to be the best evidences. Meta-analyses are the most common form of quantitative evaluation. Compare with traditional literature review, meta-analyses can improve the power of test by summarizing results and increasing sample size, and thus closer to reality (Hernandez et al., 2020). In recent years, several studies had been performed to evaluate the effectiveness and safety of SLBZP in treatment of UC by meta-analyses (Wu et al., 2017; Yang et al., 2018). For example, a meta-analysis conducted by Xiangtao Wen et al., included thirteen randomized controlled trials (RCTs) containing of 659 UC patients treated with SLBZP therapy and 598 patients treated with western medicine. The results showed the effectiveness of SLBZP was higher than that of western medicine (RR = 1.17, 95%CI [1.13, 1.22], *p* <0.001), could significantly improve time of diarrhea (RR = −12.32, 95%CI [−14.27, −10.37], *p* < 0.001), abdominal pain (RR = −8.06, 95%CI [−9.88, −6.24], *p* < 0.001), sepsis (RR = -9.89, 95%CI [−10.77, −9.00], *p* <0.001), and fever (RR = −8.29, 95%CI [−9.59, −6.98], *p* <0.001), and significantly decrease the adverse events (RR = 0.06, 95%CI [0.01, 0.40], *p* = 0.004) (Wen et al., 2017). In addition, another meta-analysis had been conducted by Yin et al., included seventeen RCTs with a total sample of 1263 UC treated with mesalazine (control group) and SLBZP combined with mesalazine (test group) (Yin et al., 2021). Results indicated that the total effective rate of patients in the combination group was higher than that in the mesalazine group (OR = 2.03, 95%CI [1.60, 2.58], *p* <0.05). And the level of IL-17 (MD = −88.29, 95%CI [-100.37, −76.21], *p* <0.00001), IL-23 (MD = −115.34, 95%CI [−130.69, -99.99], *p* <0.00001), TNF-α(MD = −10.64, 95%CI [−11.65, −9.64], *p* <0.00001), ESR (MD = −8.22, 95%CI [−9.31, −7.12], *p* <0.00001), and CRP (MD = −6.74, 95%CI [−9.99, −3.48], *p* <0.00001) in the combination group were significantly lower than those in the mesalazine group. Based on these findings, SLBZP or SLBZP combined with mesalazine proved superior to mesalazine in treating UC, and can also reduce inflammatory factors in UC. However, more large-sample-size double-blind RCTs shall be included to support this conclusion.

## 4 Studies assessing the mechanism of SLBZP in the treatment of UC

SLBZP helps prevent and treat UC by exerting immunomodulatory and antioxidant effects, repairing the intestinal mucosal damage, protecting the gut mucosa barrier, regulating relative signal pathways (including MAPK signaling pathway, toll-like receptors (TLR)/NF-κB signaling pathway, JAK/STAT signaling pathway, endoplasmic reticulum stress, autophagy pathway, and pyroptosis), regulating the balance of gut microbiota, promoting the targeting of BMSCs to the colonic mucosa, and regulating the levels of AQPs.

### 4.1 Immunomodulatory effects

#### 4.1.1 Immune cells

Immune cells, including cells of the innate and adaptive immune response, play a critical role in the processes of UC. In the case of innate immune response cells, dendritic cells (DCs) and macrophages have an important role in UC development (Kałużna et al., 2022). Antigen-presenting cells like macrophages and DCs, express a diverse repertoire of pattern recognition receptors, such as Toll-like receptors (TLRs), which play critical roles in the development of immune responses. So far, ten TLRs (TLR1-TLR10) have been identified in humans, which can recognize pathogens such as bacteria, viruses, LPS and endogenous DNA or RNA (Vijay, 2018). The mutation and maladjustment of TLRs is a key factor underlying susceptibility to UC(Kordjazy et al., 2018). According to some studies, higher levels of TLR2 and TLR4 mRNA and protein were observed in UC patients, suggesting that these receptors may play an important role in the pathogenesis of this disease (Fan and Liu, 2015; Kobayashi et al., 2020). Meanwhile, TLRs except for TLR3 activate the adaptor myeloid differentiation factor 88 (MyD88), resulting in NF-κB activation, cytokine secretion and inducing DCs maturation; these cytokines further activate TLRs/NF-κB signal pathway, which in turn further aggravate the inflammatory response (Dalod et al., 2014; Ungaro et al., 2017). Several studies showed that SLBZP efficiently inhibited the expression of TLRs and contributed to alleviate UC-induced inflammation. For example, the UC model mice (Kunming mice, SPF, male, weighing 34 ± 2 g) were induced by 3% dextran sodium sulfate (DSS), and oral administration with mesalazine (0.4 g/kg), SLBZP (31.2, 15.6, 7.8 g/kg, powder of SLBZP was dissolved in saline, the dose expression is equivalent to the weight of the original medicine, medicine, the dosage expression of SLBZS in the following is the same as this), and vehicle (water) once a day for 14 days. The results indicated that SLBZP could improve the symptoms of UC rats by decreasing the levels of TNF-α and MIF in serum, inhibiting the expression of TLR4 and NF-κB protein in colon tissue, and increasing the IL-10 and EGF levels (Sun et al., 2020a). Further, rats (Wistar rats, SPF, weighing 200 ± 20 g) were randomly divided into five groups: normal group, UC model group, SLBZP group, SLBZS + TLR2 agonist (Pam3csk4) group, and SLBZS + TLR2 antagonist (T2.5) group. All except the normal group were induced by environment and diet intervention combined with composite trinitrobenzene sulfonic acid (TNBS), ethanol in enema. Rats in normal group and UC model group orally given vehicle (0.9% sodium chloride injection, 10 ml/kg). Rats in SLBZP group orally given SLBZS (15.6 g/kg, concentrated water decoction). Prior to the oral administration with SLBZS (15.6 g/kg), rats in SLBZS + TLR2 agonist group and SLBZS + TLR2 antagonist group received respectively TLR2 agonist dose of 50 μg/mice and TLR2 antagonist dose of 2.4 μg/mice *injected* IV via the tail vein. Results showed that the IL-6, IL-1β, and TNF-α levels, the protein and mRNA expression of TLR2, MyD88, and COX-2 of UC rats were significantly increased in the UC model group; the levels significantly decreased in UC rats treated with SLBZP and TLR2 antagonist (Li et al., 2021a).

Macrophages also show significant functional differences depending on the tissue environment. During inflammation, the cytokines responsible for macrophage activation are secreted, and depending on the activation method, macrophages can be divided into classically activated (M1) or alternatively activated (M2). A murine model of experimental colitis showed that DSS increased the proportion of P1 peritoneal macrophages, which was restored by SLBZP (1.8, 3.6 g/kg) treatment. Moreover, a co-culturing system was established to decipher the interaction between BMDMs and NCM460 cells treated with TNF-α and/or SLBZP serum (the serum was obtained from colitis mice subjected to 3.6 g/kg SLBP treatment). Consistently, the proportion of P2-P4 macrophages was higher in the SLBZ group, concomitant with a decreased migration capacity, implying the transition to M2 macrophages (Yu et al., 2022).

**FIGURE 3:**
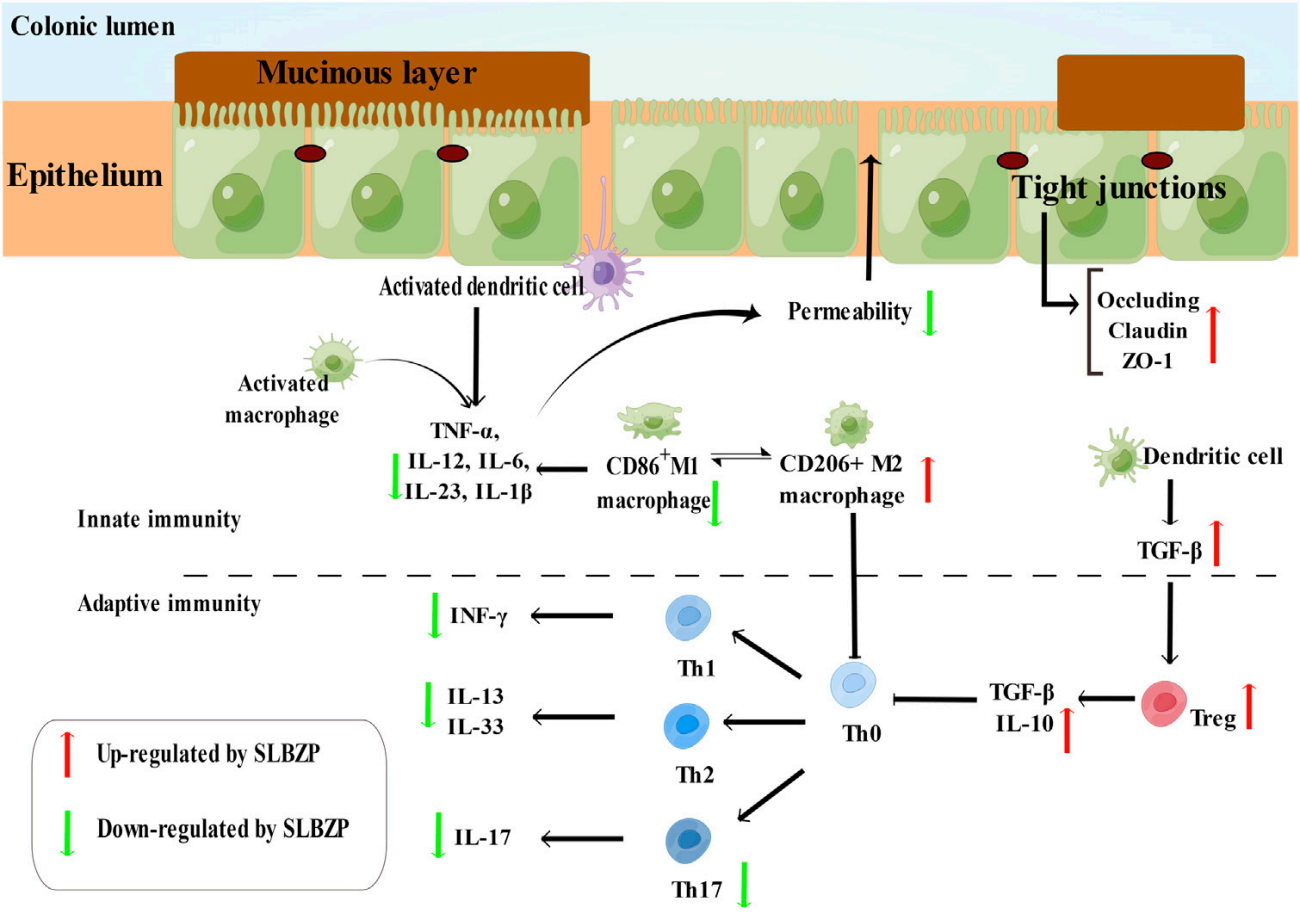
Regulation of Immune functions and intestinal permeability of SLBZP in UC.

Unlike the innate immune system, the adaptive immune system must be activated before a specific immune response can occur. Upon being stimulated by inflammatory cytokines, the naive T cells begin to differentiate into different lineages, including Th cells, Th1 cells, Th2 cells, Th17 cells, and Treg cells, which play a key role in the development and progression of UC. Depending on the expression of the CD4 and CD8 cell surface molecules, lymphocytes can be divided into T CD8^+^ (mainly cytotoxic cells) and T CD4^+^ cells (Rabe, et al., 2019). UC model mice (Kunming mice, SPF, male, weighing 20 ± 2 g) were induced by TNBS, and oral administration with dexamethasone (1 mg/kg), SLBZP (1.4, 2.8, 5.6 g/kg, powder of SLBZP was dissolved in saline), and vehicle (water) once a day for 10 days. Results indicated that high dosage of SLBZP (5.6 g/kg) could significantly increase the serum IL-10 level and the ratio of CD4^+^, CD25^+^, and Foxp3^+^ cells to CD4^+^ T cells and decrease serum IL-1β and TNF-α levels, in turn, treating UC mice (Li et al., 2014b). Further, a study showed that SLBZP (7.5, 15, 30 g/kg, concentrated water decoction) could regulate the balance of Th17/Treg and restore the immune function of UC rats by downregulating the levels of IL-17, IL-23, IL-6, TNF-α and upregulating the levels of IL-10 (Li et al., 2017). Another study also showed that after 21 days treatment of SLBZP (6, 12, 24 g/kg, concentrated water decoction), high dose of SLBZP could ameliorate the symptoms of UC rats by regulating the balance of Th17/Treg and decreasing the expression of c-fos in colon tissue (Yu et al., 2018). In addition, UC model rats were induced by TNBS, and oral administration with mesalazine (0.4 g/kg), SLBZP (11.3, 22.6, 45.2 g/kg, concentrated water decoction), and vehicle (0.9% sodium chloride injection) once a day for 14 days. Results showed that middle dosage and high dosage of SLBZP had a therapeutic effects on UC, and it was related to the regulation of expression of RORγt/FoxP3 and correction of the imbalance of Th17/Treg (Qi et al., 2022). As shown in Figure 3.

#### 4.1.2 Inflammatory factors

The imbalance of pro-inflammatory and anti-inflammatory cytokines remains one of the key factors causing UC (Xu et al., 2016). Lamina propria macrophages and T cells are activated when the intestinal mucosal barrier is damaged. These secrete pro-inflammatory cytokines such as IL-1, IL-6, IL-17, IL-23, and TNF-α. Meanwhile, these pro-inflammatory cytokines stimulate immune cells to secrete more pro-inflammatory cytokines. Ultimately, the balance of pro-inflammatory and anti-inflammatory cytokines is disturbed, leading to a cytokine storm, and causing continuous inflammation of intestinal mucosa and ulcer (Isidro et al., 2014). Studies showed that SLBZP (1.4, 2.8, 5.6 g/kg, powder of SLBZP was dissolved in saline) could downregulate TNF-α and IL-1β levels and upregulate IL-10 levels in the colon tissue of TNBS-induced rats (Li et al., 2014a). Another study indicated that the levels of IL-6, IL-8, TNF-α, and the expression of NF-κB and p65 increased significantly in UC rats; these levels decreased significantly in rats treated with SLBZP (12 g/kg, concentrated water decoction) (Li et al., 2015a). Additionally, a large amount of literature also suggests that SLBZP affects the levels of other cytokines (IL-3, IL-13, IL-33, TGF-β, IL-4, IL -17, IL-23) (Li et al., 2015b; Bi et al., 2017; Chen et al., 2018a; Ding et al., 2018), and the major cytokines involved in SLBZP treatment for UC are shown in Supplementary Table S1.

### 4.2 Gut mucosa barrier

The gut mucosal barrier, consisting of mechanical, chemical, immunological, and biological barriers, is an important system of intestinal defense and maintains the integrity of the intestinal barrier and gut homeostasis by isolating the harmful elements. The mechanical barrier includes intestinal mucosa epithelial cells (ICEs) covered with mucosal layer and intercellular tight junctions (with components such as occludin, claudin, and ZO-1) and is the structural foundation for protecting against pathogen invasion and maintaining the intestinal permeability. Intestinal lymph and secretion immune proteins, resident flora, glycoproteins, and digestive juices secreted by ICEs, respectively, are the components of chemical, immunological, and biological barriers. Research has confirmed that mechanical, chemical, immunological, and biological barriers are involved in the pathogenesis of UC (Actis et al., 2014). When the gut mucosa barrier is damaged in UC, many inflammatory factors are produced. These factors can not only induce the apoptosis of ICEs, but can also influence the expression and distribution of tight junction proteins through MLCK and PKC signaling pathways, damage the structure of the mechanical mucosal barrier, and induce intestinal immune response (Actis et al., 2014).

One study found that SLBZP SLBZP (3, 6, 12 g/kg, concentrated water decoction) could help heal the tight junction of UC mice by increasing the expression of colonic components such as occludin, claudin, ZO-1, as well as JAM gene and protein (Liu et al., 2015). Another study showed that the expression levels of occludin in the colonic tissue were significantly downregulated and expression levels of P65, MLCK, MLC2, P-MLC were significantly upregulated in UC mice. Following treatment with sulfasalazine (0.52 g/kg) and SLBZP (7.8, 15.6, 31.2 g/kg, concentrated water decoction), these indicators improved significantly, in particular, high dosage of SLBZP (Liu et al., 2018a). These results indicated that SLBZP downregulates the expression of occludin, claudin, ZO-1, and JAM protein in the colon tissue, thus maintaining the normal permeability of the intestinal mucosa and repairing the intestinal mucosal damage, which may involve inhibition of the MLCK/MLC signaling pathway. Additionally, extracellular matrix degradation can increase intestinal permeability and decrease the barrier function of the intestinal mucosa, which plays an important in the pathogenesis of UC (Kirov et al., 2019). Matrix metalloproteinases (MMPs) are proteolytic enzymes that degrade ECM proteins. The pro-inflammatory factor can stimulate the proteolytic enzymes, and activated MMPs can further aggravate the inflammatory response of the intestinal tract (Bai et al., 2020). MMP-2 and MMP-9 are the major gelatinases among MMPs, which can degrade collagen type IV to prevent cell infiltration and inflammatory proliferation. A clinical study indicated that SLBZP (1 dose per day, decocted in water, twice daily) could improve the symptoms of UC patients, and the mechanism may involve a decrease in the expression of MMP-2 and MMP-9 (Wang, 2016).

### 4.3 Mesenchymal stem cells and cell adhesion molecules

Repair and reconstruction of the damaged colonic mucosa are central to UC treatment. The repair and reconstruction of colonic mucosa rely primarily on colonic mucosa stem cells that can differentiate into mature colonic mucosa cells (He et al., 2012b). Bone mesenchymal stem cells (BMSCs) have good immune regulatory effects and homing feature that promotes migration to the injury site, adhesion, and colonization, in turn repairing and reconstructing the colonic mucosal tissue of UC (Zheng and Wang, 2022). The differentiation, growth, migration, and homing of BMSCs are regulated by a combination of various factors, including several chemokines and adhesion molecules (such as ICAM-1, VCAM-1, and VLA-4) (He et al., 2012a). The study revealed that inflammatory damage to UC could promote homing of BMSCs to colon tissue; however, it did not play a role in the repair and regeneration of the tissue injured due to UC. Research indicated that SLBZP (22.6 g/kg, concentrated water decoction) could not only promote BMSCs homing to colon tissue but also help repair and regenerate tissue injured due to UC, which may involve the enhancement of the SDF-1/CXCR4 signaling pathway (Cui et al., 2020). Meanwhile, studies have shown that SLBZP (22.6 g/kg, concentrated water decoction) could promote the proliferation and migration of BMSCs and increase the adhesion properties by regulating the expression of VCAM-1 and VLA-4 (Liu et al., 2018b; Shi et al., 2018a).

### 4.4 Antioxidant effect

Reactive oxygen species (ROS) and reactive nitrogen species (RNS) are produced during oxygen metabolism, and low-to medium-concentration ROS and RNS are molecular signals of mitogenic response or defense responses against invasion by pathogens; excessive expression of these factors can induce oxidative stress (Li et al., 2020a). Oxidative stress altering the inflammatory response causes damage to lipids, proteins, and DNA, and also results in cell apoptosis and cancer cell transformation, which is potentially dangerous. Research showed that oxidative stress is a key factor involved in the progression of many diseases such as UC (Piechota-Polanczyk and Fichna, 2014). Antioxidants inhibit the process of cell oxidation or scavenge ROS, such as free radicals. When antioxidants are inadequate or exhibit lower activity, oxidant molecules can prevail, disrupting the cell functions and leading to cell death. The endogenous cellular antioxidant defense system consists of enzymatic antioxidants (SOD, CAT, GSH-Px, and GR) and non-enzymatic antioxidants (antioxidant vitamins, trace elements, coenzymes, and cofactors) (Wang et al., 2019). Abnormal free radical metabolism is generally observed in UC patients, and the ROS can induce excess lipid oxidation in UC, exacerbating the damage to intestinal mucosa (Pavlick et al., 2002). Therefore, maintaining the oxidant/antioxidant status balance is critical to treating UC and SOD, and MDA plays a significant role in balancing the system. SOD represents the ability to scavenge free radicals and can inhibit the response of excess lipid peroxidation. MDA levels reflect the degree of lipid peroxidation in the body and indirectly reflect the degree of cellular damage (Deng et al., 2016). In a study UC model rats were induced by environment and diet intervention combined with composite TNBS and ethanol, and oral administration with sulfasalazine (0.5 g/kg), SLBZP (12 g/kg, concentrated water decoction), and vehicle (0.9% sodium chloride injection) once a day for 14 days results showed that SLBZP could increase the SOD levels and decrease MDA levels, subsequently leading to clinical benefits in UC rats (Li et al., 2012). In another study, UC model rats were treated with SLBZP (0.472, 0.945, 1.89 g/kg, granule of SLBZP was dissolved in water). Compared with the control group, the levels of PCT, CRP, EPO, and HIF-1α were significantly upregulated, and levels of iNOS, MPO, SOD, and MDA were significantly downregulated in the model group. Compared with the model group, the levels of PCT, CRP, EPO, and HIF-1α were significantly downregulated, and levels of iNOS, MPO, SOD, and MDA were significantly upregulated in the SLBZP group, in particular, high dosage SLBZP group (Xiong et al., 2021).

### 4.5 Regulation of signaling pathways

#### 4.5.1 MAPK signaling pathway

MAPK is a highly conservative signal-transducing module in eukaryotic cells and is an important member involved in the interaction between the inner and outer in cell reaction, which can mediate extracellular signals stimulation to intracellular and regulate the progress of cell growth, differentiation, migration, and inflammation. The MAPK family is composed of the extracellular regulated kinase (ERK) 1/2, JNK, and p38 MAPK. MAPK pathway can be activated by several stimuli such as inflammatory cytokines, growth factors, and cellular stress. MAPK pathway can activate the c-Jun and c-fos through a cascade of ERK, JNK, and p38 MAPK, which regulate the expression of inflammatory cytokines including IL-1, TNF-α, and IL-6 and contribute to intestinal mucosal inflammation (Jing et al., 2019). In an animal experiment, UC rats were induced by the environment and diet intervention combined with composite TNBS and ethanol in enema and treated with SLBZP (12 g/kg, concentrated water decoction). ERK and p38MAPK protein expression were significantly increased in the model group, while SLBZP could reserve those indicators (Li et al., 2013). Another experiment showed that the expression of p38 MAPK, and TNF-α levels were significantly downregulated, and IL-4 protein of UC rats were significantly upregulated in the SLBZP group (24 g/kg, concentrated water decoction) (Bi et al., 2017). These findings indicated that upregulation of IL-4 concentration as well as downregulation of TNF-α concentration by the MAPK pathway might be a part of the mechanism of SLBZP to treat UC.

#### 4.5.2 TLRs/NF-κB signaling pathway

Studies have confirmed that the expression of relative genes (TLR4, MyD88, NF-κB), mRNA, and proteins from the TLR/MyD88-dependent pathway were significantly upregulated in UC. In contrast, negative regulation of the TLRs/NF-κB signaling pathway was effectivein alleviating UC clinical syndromes (Li et al., 2019). Under normal physiological conditions, NF-κB is inhibited by binding IκB and is retained in the cytoplasm. Upon cellular stimulation, the active signal of NF-κB can activate the IKK to induce phosphorylation and degradation of IκB, so as to prevent the inhibition of NF-κB by IκB. Thus, NF-κB protein when activated, facilitates the transcription and expression of downstream genes, playing a regulatory role in body immunity, cell inflammatory, cell survival, cell growth, cell differentiation, and apoptosis (Wullaert et al., 2011). Studies also showed that SLBZP could ameliorate clinical syndromes of UC by upregulating serum IL-10 and EGF levels, downregulating serum TNF-α and MIF levels, and the expression of TLR4 and NF-κB proteins (Sun et al., 2020b). Another study indicated that SLBZP (15.6 g/kg, concentrated water decoction) could decrease the levels of serum TNF-α, IL-6, IL-1β, and the expression of TLR2, MyD88, COX-2 mRNA and proteins in UC rats (Li et al., 2021b). Moreover, SLBZP (15.6 g/kg, concentrated water decoction) could decrease the concentrations of IL-17, IL-23, IL-6, TNF-α, and IL-1β, decrease the expression of NF-κB p65, IκKβ, and increase the expression of IκBα protein in the UC (Chen et al., 2018b; Li et al., 2020b). These results indicated that SLBZP could reduce the inflammatory response through the negative regulation of the TLRs/NF-κB signaling pathway, which might be an important mechanism through which SLBZP helps treat UC.

#### 4.5.3 JAK/STAT signaling pathway

**FIGURE 4:**
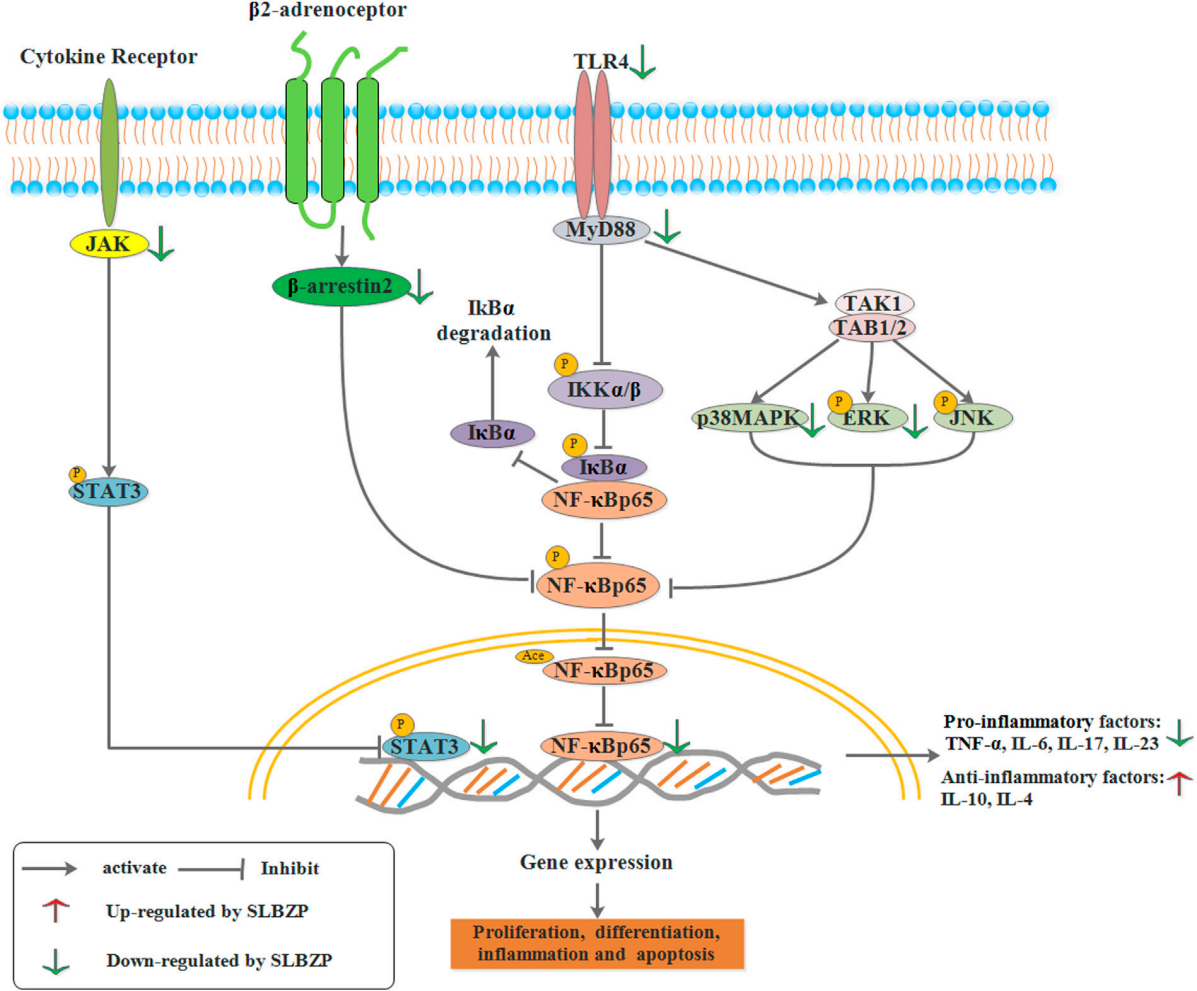
Relative cell signal transduction including MAPK signaling pathway, TLR/NF-κB signaling pathway, JAK/STAT signaling pathway were regulated by SLBZP.

JAK/STAT signaling pathway consists of tyrosine kinase-associated receptor, JAK/STAT, and tyrosine kinase-coupled receptors is a common pathway underlying numerous cellular signal transduction pathways. It plays an important role in physiology and pathology, including immune defense, cell differentiation, cell growth, cell apoptosis, and tumorigenesis (Xin et al., 2020). The JAK family includes JAK1, JAK2, JAK3, and TYK2. JAK1, JAK2, and TYK2 are present in various cells and tissues, while JAK3 only exists in the bone marrow and lymphatic system. STATs, a family of latent cytoplasmic proteins, are the substrate of JAK, which can couple with the signaling pathway of tyrosine phosphorylation, thus exerting biological effects through transcription control (Xin et al., 2020). JAK plays a role in the inflammatory response, while JAK inhibitor can relieve the UC to some extent, a potential therapeutic approach to treat UC (Cordes et al., 2020). According to current research, STAT3 is known to be related to colonic inflammation and activated by different cytokines and growth factors (Jin et al., 2017). Increased STAT3 phosphorylation at tyrosine residues is found in the UC model induced by DSS and in the epithelial tissue and lamina propria cells of UC patients (Aggarwal et al., 2009). Animal experiments showed that the concentration of serum IL-6, and the expression of STAT3 and JAK2 protein in SLBZP (12, 24 g/kg, concentrated water decoction) and mesalazine (0.3 g/kg) groups were significantly lower than those in the model group. In comparison, the concentration of serum IL-10 was significantly higher than that in the UC model group. It showed that the mechanism of SLBZP in treating UC might involve inhibition of the JAK/STAT3 signal pathway (Tong, 2021). As shown in Figure 4.

#### 4.5.4 Endoplasmic reticulum stress pathway and autophagy

The endoplasmic reticulum (ER) is an important subcellular organelle involved in protein synthesis, post-translational modification, and proper folding. When the intracellular environment changes, the endoplasmic reticulum stress (ERs) is activated to combat the protein misfolding and synthesis damage through multiple ways. While holding the balance of the endoplasmic reticulum, excessive ERs will induce cell apoptosis. ERs alleviate the cell burden in two ways: they strengthen the ability of folding and processing to relieve the accumulation of protein and also relieve the synthesis of new protein. The process is finished by unfolded protein responses (UPR) that can activate the unfolded protein receptors, including IRE1, PERK, and ATF-6. Under the normal conditions, IRE1, PERK, and ATF-6 exhibit an inactive state by binding to the glucose-regulated protein 78 (GRP78). UPR can disaggregate the three proteins from GRP78, thus receiving and inhibiting ERs through the IREl-XBP1 pathway, PERK-el F2α-ATF-4 pathway, and ATF-6 pathway. In addition, the CHOP pathway, IRE1-TRAF2-ASK1 pathway, and caspase pathway are pathways mediated by ERs and lead to cell apoptosis (Yap et al., 2021). ERs as a regulation mechanism widely exist in the body, which similarly plays an essential role in the pathological process of IBD (Bogaert et al., 2011). Research has shown that the expression of GRP78 and XBP1 increased significantly in UC patients (Shkoda et al., 2007; Tréton et al., 2011). ERs can activate NF-κB to promote the inflammatory response, which can also induce ERs by active oxygen and TNF-α. A study found that phosphorylation of GRP78, IRE1, and the levels of CHOP were upregulated, and the cell apoptosis was increased in ATF6α−/− mice, ultimately exhibiting severe damage of intestinal epithelial mucosa (Brandl et al., 2009). In cell and animal experiments, ERS of IEC-6 cells and UC mice were induced by LPS, and 5% DSS, respectively. The former was administered with the serum containing SLBZP (the serum was obtained from colitis mice subjected to 3.6 g/kg SLBP treatment), and the latter was administered with SLBZP (3, 6, 12 g/kg, concentrated water decoction). The results indicated that the expression of GRP78, IRE1, P-IRE1, PERK, pJNK, P-eIF2α, and CHOP proteins were significantly increased in ERS of IEC-6 cell and UC mice while they decreased significantly in ERs of IEC-6 cell and UC mice treated with SLBZP (Xu, 2020). This suggests that SLBZP could regulate the ERs level of colon tissue through ERs pathways such as IRE1-XBP1, PERK -EIF2α, and ATF-6 pathways, to alleviate intestinal injury in UC.

**FIGURE 5:**
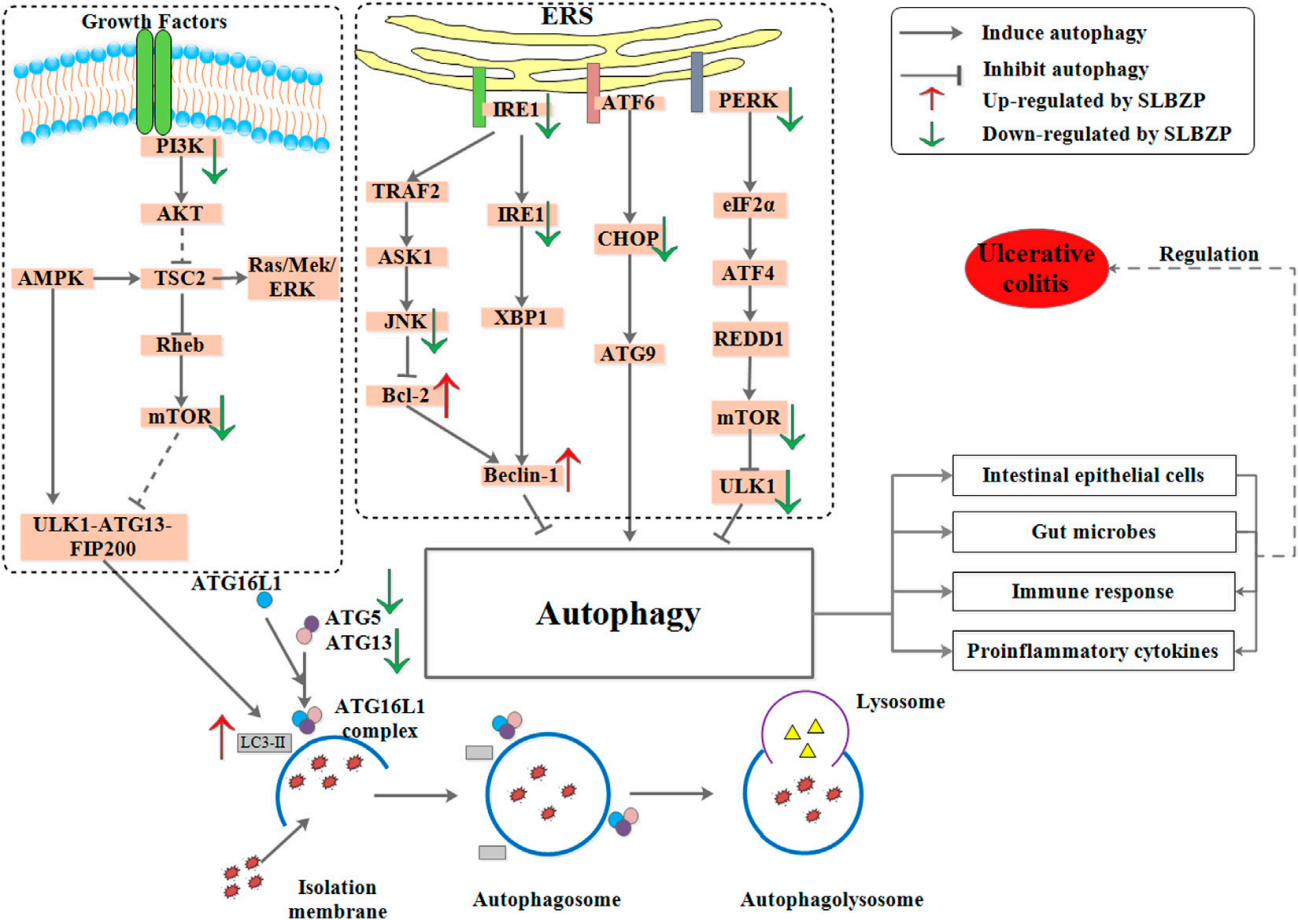
Autophagy and endoplasmic reticulum stress were regulated by SLBZP.

Autophagy involving the formation of autophagosomes, fusion with lysosomes, and degradation, is a ubiquitous phenomenon in eukaryotic cytoplasmic and plays a role in maintaining the cell survival and update, re-utilizing materials, and maintaining cellular environmental homeostasis (Zhao and Zhang, 2018). Autophagy-related genes (ATG), such as ATG5, ATG6, ATG8, and ATG12, play a very important role in autophagy (Lee and Tournier, 2011). The PI3K/Akt/mTOR pathway is a classical signal pathway involved in the regulation of autophagy. The PI3K is divided into three types: I, II, and III. The activation of PI3K type Ⅰ can activate the downstream signaling pathway to block autophagy. Beclin1 (ATG6) activation by PI3K type III is an important step in the initiation of autophagy. p62 is an autophagic substrate protein that can bind to LC3, forming a complex followed by autophagic degradation (Liu et al., 2018c). LC3 converts to LC3-I and distributes in autophagic vesicle and autophagosome under normal conditions. When autophagy is induced, LC3-I is modified to LC3-II, which is integrated into the autophagosome membrane (Liu et al., 2016). In the IBD pathologic process, damage to the intestinal epithelial barrier and increased mucosal permeability lead to swelling and alimentary deficiency of intestinal epithelial cells, inhibiting autophagy (Liu et al., 2016). In a cell-based study, IEC-6 injury induced by LPS was treated with the serum containing SLBZP (the serum was obtained from rats subjected to 41.6 g/kg SLBP treatment). Results showed that the concentrations of IL-1β, IL-8, and the expression of ATG5, ATG13, and ATG16 mRNA were significantly increased, and the level of IL-10 was significantly decreased in the model group compared with the blank group. These indicators were reversed in the SLBZP group, indicating that SLBZP could induce the inflammatory damage to IEC-6 cells through the autophagy pathway (Liu et al., 2019). In another animal experiment, UC was induced in mice by dosing with 5% DSS, and oral administration with mesalazine (2 g/kg), rapamycin (4 mg/kg), SLBZP (3, 6, 12 g/kg, concentrated water decoction), and vehicle (water) once a day for 15 days. The results showed that SLBZP could ameliorate UC syndrome by increasing the LC3-Ⅱ, beclin1 phosphorylation, and 4EBP protein expression and inhibiting PI3K, mTOR, p-p62 phosphorylation, and ULK1 protein expression. These results were observed in mesalazine group and rapamycin group. The above indicated that SLBZP helps treat UC by regulating the phosphorylation of PI3K, mTOR, and p-p62 proteins in the autophagy pathway of intestinal epithelial cells (You et al., 2019). These findings indicate that SLBZP regulates the ERs and autophagy signaling pathways, thus contributing to healing in UC (As shown in Figure 5).

#### 4.5.5 Pyroptosis

Pyroptosis is a caspase1-dependent (classical pathway) and caspase11, 4/5-dependent (non-classical pathway) pathway of programmed cell death. It is characterized by the cytoplasmic membrane rupture in a short period and the release of cellular content and pro-inflammatory mediators, including IL-1β, IL-18, and HMGB-1 (Walle and Lamkanfi, 2016). The pyroptosis process relies on caspase-1, -11, -4/5, vesicular shedding, and the cleavage of proteins like GSDMD that create pores in the cell membrane, which leads to cellular rupture and discharge of contents under osmotic pressure and cell membrane movement (Ding et al., 2016). Caspase-11 cleaves GSDMD and generates amino-terminal fragments, increasing the reliance on caspase-1 for pyroptosis and NLRP3 dependence in a cell-intrinsic manner. GSDMD-N is considered a key target of caspase-11 and a critical mediator of the host to gram-negative bacteria (Kayagaki et al., 2015). Demon D et al. found that caspase-1, -11, -4/5, and NLRP were highly expressed in intestinal cells and positively correlated with the severity of inflammation in UC(Demon et al., 2014). LPS from the wall of gram-negative bacteria could enter the cell, specifically activating the reliance on caspase 11 and IL-18 secretion of pyroptosis, resulting in cell death. Similarly, both caspase 1 and caspase 11 could lead to cell death. These results showed that caspase-11 and GSDMD-N drove the pro-immunogenic cell death signal (Shi et al., 2014). Animal experiments showed that SLBZP (1.18 g/kg, powder of SLBZP was dissolved in saline) could significantly decrease the levels of serum IL-18, TNF-α caspase-1, caspase-11, and the expression of GSDMD-N and NLRP3 protein in UC mice induced with DSS. These results indicated that the mechanism of SLBZP was related to the regulation of classical (caspase-1) and non-classical (caspase-11) pathways in pyroptosis, in which the non-classical (caspase-11) pathways may play a significant role (Li, 2019).

### 4.6 Aquaporins

The expression of aquaporins (AQPs) in the intestinal tissue is closely related to UC pathogenesis. Studies indicated that the low expression of AQPs was observed in the early stage of UC before the appearance of intestinal epithelial injury. AQPs are distributed extensively in the intestinal tract and play an important in regulating water transport, permeability, secretion, and absorption of fluid in the intestinal tract (Cohly et al., 2008). Evidence showed that inhibiting the expression of AQP3 and AQP4 could cause diarrhea, leading to an inflammatory response, ultimately resulting in UC(Hardin et al., 2004; Planell et al., 2013). Additionally, the MAPK pathway consists of p38MAPK, ERK, and JUK, which can regulate cell growth, differentiation, and apoptosis, and take part in the regulation of AQP3 and AQP4 (Li et al., 2015c). Animal experiments indicated that SLBZP (12 g/kg, concentrated water decoction) could increase the expression of AQP3 and AQP4 in UC, and this effect was partially inhibited by U0126 (ERK1/2 inhibitor) or SB203580 (p38MAPK inhibitor). This suggests that SLBZP can ameliorate UC by increasing the expression of AQP3 and AQP4 through ERK/P38MAPK pathway (Li et al., 2015a).

### 4.7 Regulation of gut microbiota

Gut microbiota has important immune, metabolic, and intestinal protective functions. Moreover, gut microbiota can inhibit the growth of potentially pathogenic bacteria by producing antibacterial factors and colonization resistance. Gut microbiota in healthy individuals is in a dynamic equilibrium state. In contrast, UC patients or mice showed imbalance, manifested as the abundance of enteropathogenic bacteria and lack of beneficial bacteria in the intestinal tract (Yu, 2018). Studies have shown that *Escherichia/Shigella* and especially *Escherichia coli*, which belongs to the family Enterobacteriaceae, are enriched in patients or mice with UC. At the same time, firmicutes are in reduced quantity, particularly Blautia, Clostridium, Coproccocus, and Roseburia (Bian et al., 2020). In recent years, regulation of gut microbiota for UC treatment has added a new therapeutic strategy and has increased the possibility of curing UC patients (Chen et al., 2014). Animal experiments have shown that high dosage of SLBZP (24 g/kg, concentrated water decoction) could increase Prevotella and Oscillospira that produce SCFAs and decrease the opportunistic pathogens, including Desulfovibrio and Bilophila, which reduce the diversity of gut microbiota and increase abundance (Gu et al., 2021). Another clinical experiment showed that SLBZP combined mesalamine with (6 g of SLBZP particles three times a day; 6 g of mesalamine a day for 8 weeks) could improve the clinical syndrome of UC by regulating the gut microbiota and increasing the microbial levels of tryptophan metabolites, including indole-3-propionic acid and indole-3-acetic acid (Jiao et al., 2022). Only a few studies have assessed the effect of SLBZP on gut microbiota, and more in-depth and comprehensive research on gut microbiota and SLBZP in UC is needed in the future.

## 5 Pharmacological studies of a single Chinese herb and its active ingredients of SLBZP on UC

**FIGURE 6:**
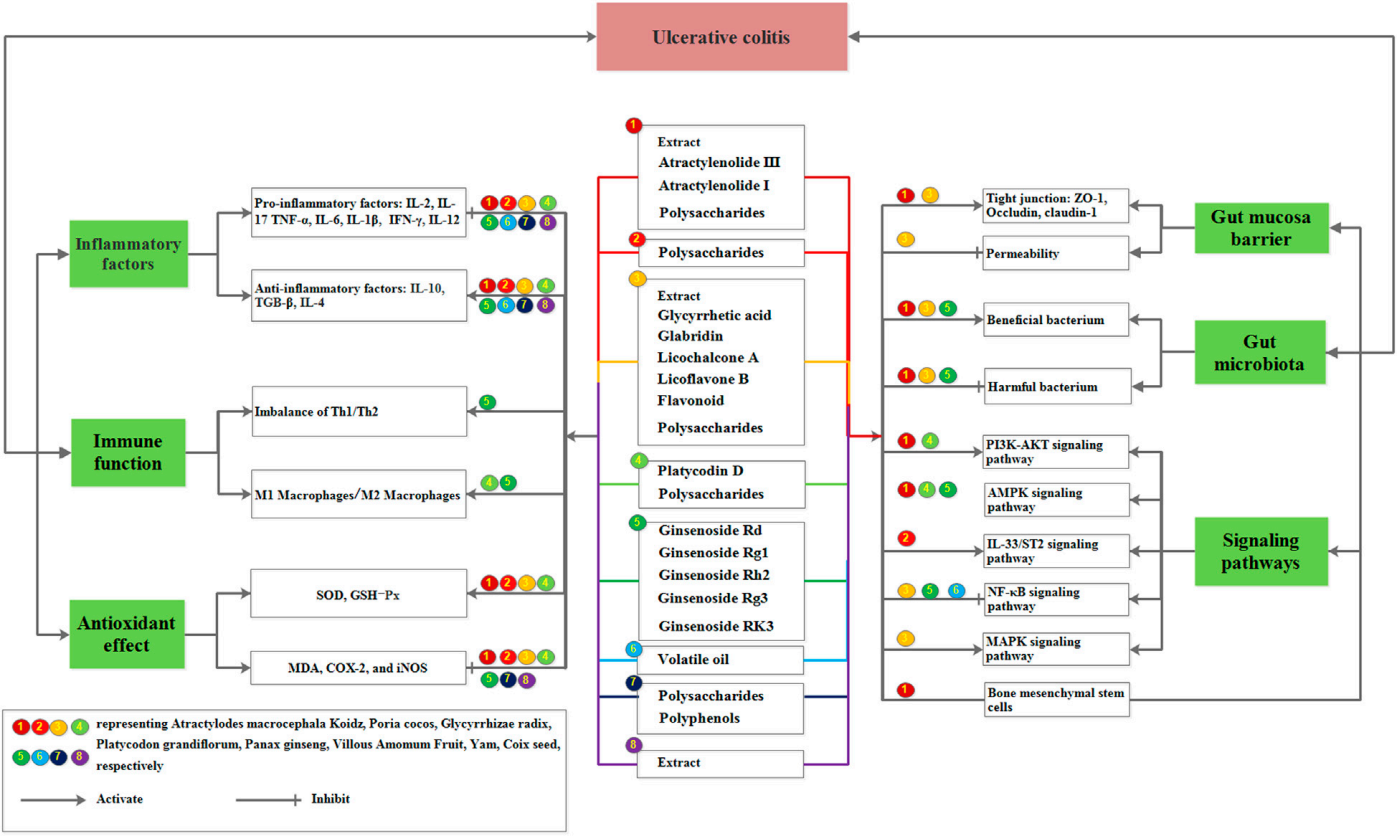
Effects of single Chinese herb and its active ingredients from SLBZP in treatment of UC.

**FIGURE 7:**
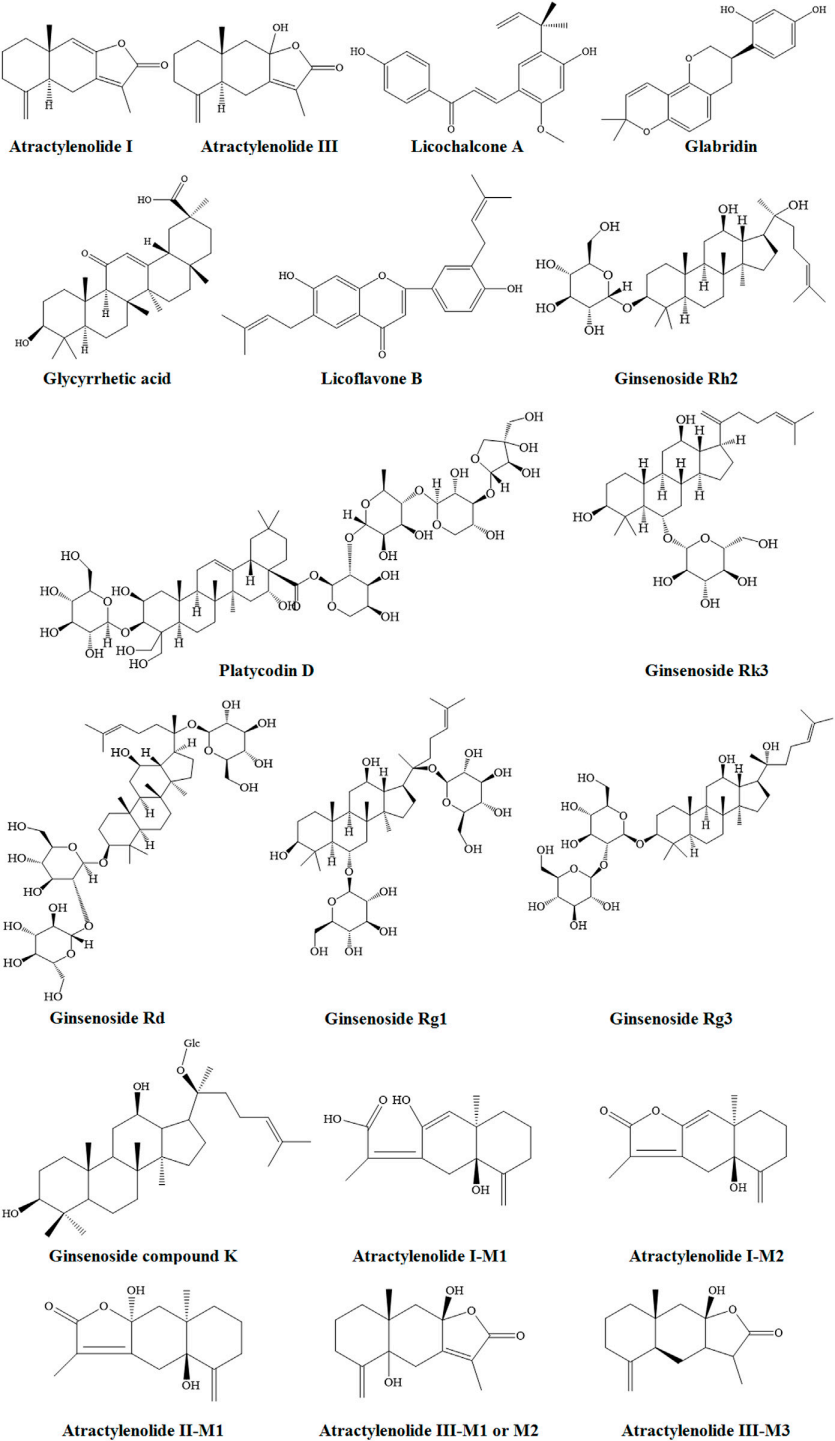
The structures of prototype and metabolites components of herbs in SLBZP.

SLBZP contains 10 Chinese drugs derived from roots, rhizomes, seeds, seed kernels, fruits, and sclerotium. Over the years, the active components of these Chinese drugs, including triterpenoids, polysaccharides, volatile, flavonoids, alkaloids, and organic acids, have been studied by various researchers. The specific attributes of drugs are shown in Supplementary Table S2, and their mechanisms are summarized in Figure 6. The structures of prototype and metabolites components of herbs in SLBZP are shown in Figure 7.

### 5.1 *Atractylodes macrocephala* Koidz. (Bai Zhu)

*Atractylodes macrocephala* Koidz., a common drug in TCM, possesses the effects of tonifying *qi* and strengthening the spleen. It is used to cure patients with splenic asthenia, anorexia, edema, excessive perspiration, and abnormal fetal movement. Modern research showed that the major constituents of *Atractylodes macrocephala* Koidz. were sesquiterpenes, polyacetylenes, polysaccharides, and organic acids, exhibiting pleiotropic biological activities, including anti-inflammatory, anti-tumor, regulation of gastrointestinal function and immune function (Yao et al., 2019). In recent studies, *Atractylodes macrocephala* Koidz. and its constituents have demonstrated potential efficacy in different experimental models of UC. A study reported that the water extract of *Atractylodes macrocephala* Koidz. (10 g/kg, concentrated water decoction) could protect against the acetic acid and dinitrochlorobenzene-induced colitis in a rat model by regulating the levels of IL-2, IL-10, and IL-17 cytokines in serum (Zhu et al., 2014). Meanwhile, another study showed that *Atractylodes macrocephala* Koidz. (10 g/kg, concentrated water decoction) could reduce the expression of TNF-α, IL-6, and IL-1β and regulate the balance of gut microbiota to treat the DSS-induced UC rats (Ye et al., 2014a). Similarly, the active ingredients of *Atractylodes macrocephala* Koidz., including polysaccharides, atractylenolide III, and atractylenolide I, exhibited potential efficacy in treating UC mice induced with DSS. Feng et al. reported that polysaccharides from *Atractylodes macrocephala* Koidz. (10, 20, 40 mg/kg) could regulate the balance of gut microbiota and its metabolism to achieve the therapeutic effects of UC(Feng et al., 2020). Additionally, polysaccharides from *Atractylodes macrocephala* Koidz. (540 mg/kg) could not only promote BMSC homing to the injured tissue and regulate cytokines such as IL-6, IL-10, IL-17 A, and TGF-β for preventing TNBS-induced rats colitis but also promote the migration of IEC *in vitro* and influence multiple genes (Zheng and Wang, 2022). Han and colleagues reported that atractylenolide III (5, 10 mg/kg) could ameliorate DSS-induced colitis inflammatory and oxidative stress by regulating the MDA and GSH contents, SOD activity, and the expression of TNF-α, IL-6, COX-2, and iNOS mRNA. Additionally, the intestinal epithelial barrier destruction and mitochondrial dysfunction were decreased. LPS-treated IEC-6 cells and DSS-induced colitis mouse model revealed that the expression of p-AMPK, SIRT1, and PGC-1α, along with acetylated PGC-1α, was facilitated by atractylenolide III (40 and 80 μM) (Han et al., 2022). Linghang Qu et al. demonstrated that atractylenolide I (50 mg/kg) could improve the induction of mucoprotein MUC2, tight junction proteins (ZO-1, Occludin), and inflammatory factors TNF-α, IL-6, IL-1β in DSS-induced colitis mice. Meanwhile, atractylenolide I could regulate the diversity and abundance of gut microbiota and its metabolism. Furthermore, they found that two genes, SPHK1 and B4GALT2, relating to the metabolism of fructose and galactose, and the activation of the PI3K-AKT pathway, were inhibited in UC mice treated using atractylenolide I (Qu et al., 2022a).

### 5.2 *Poria cocos* (SchW.) Wolf. (Fu Ling)

*Poria cocos* (SchW.) Wolf. can promote diuresis, eliminate dampness, invigorate the spleen, and calm the heart. The polysaccharides of *Poria cocos* (SchW.) Wolf. are its major bioactivity component and account for approximately 70–90% of dry sclerotium, which has proven therapeutic activities such as anti-tumor, anti-inflammatory, and immunomodulation (Deng et al., 2020). A study reported that a carboxymethyl polysaccharide CMP33 from *Poria cocos* (SchW.) Wolf. (100, 300 mg/kg) could improve the syndrome of TNBS-induced colitis mice by regulating the MPO and MDA contents and the levels of pro-inflammatory (TNF-α, IL-6, L-1β, IL-12, IFN-γ, IL-2, IL-17) cytokines and anti-inflammatory cytokines (IL-4, IL-10). The results of proteomic and metabolomic studies showed that 2-hydroxybutyric acid-(GPT, GGH)-glutathione-ALB-testosterone-TTR-dihydrotestosterone and (PYY, FABP2, HMGCS2)-oleic acid-TTR-dihydrotestosterone were the key protein–metabolite pathways (Liu et al., 2018d). Tonger Liang et al. reported that polysaccharides from *Poria cocos* (SchW.) Wolf. (50, 100, 200 mg/kg) could protect against the TNBS-caused colitis in a rat model by decreasing the levels of IL-33, IL-5, IL-13, IL-6 cytokines and expression of IL-33 and ST2 proteins to inhibit the IL-33/ST2 signaling pathway (Liang et al., 2020).

### 5.3 *Glycyrrhiza uralensis* Fisch. (Gan Cao)

*Glycyrrhiza uralensis Fisch.* primarily contains triterpenoids, polysaccharides, flavonoids, and coumarins. Modern research has showed that *Glycyrrhiza uralensis Fisch.* has various pharmacological activities, including anti-tumor, anti-inflammatory, antibacterial, and anti-viral properties (Deng et al., 2021). An animal experiment suggested that *Glycyrrhiza uralensis Fisch.* extract (50, 100 mg/kg) is effective against DSS-induced colitis in mice. It functions by decreasing the levels of inflammatory factors, including IL-6 and TNF-α, and suppressing the expression of COX-2, NF-κB, and PGE2 proteins (Jeon et al., 2016a). Qin Lu and colleagues found that *Glycyrrhiza uralensis Fisch.* extract could inhibit apoptosis by regulating the expression of apoptotic or anti-apoptotic proteins, including muc3, BAX, muc1, bcl-2, FGF-15, P-gp, SHP, and regulating the immune function through FXR/P-gp pathway (Lu et al., 2021). In addition, triterpenoids, polysaccharides, and flavonoids from *Glycyrrhiza uralensis Fisch.* are considered to have a potential therapeutic role in UC. A study reported that glycyrrhetic acid (10, 50 mg/kg) could decrease the levels of IL-6, IL-1β, and TNF-α, and suppress the expression of COX-2, NF-κB, and PGE2 protein for treating the DSS-induced colitis mice (Jeon et al., 2016b). Nahla E. El-Ashmawy et al. reported that glabridin (50 mg/kg) exhibits anti-inflammatory and antioxidant effects in DSS-induced colitis mice by regulating the levels of TNF-a and cAMP, the activity of MPO, and gene expression of iNOS in the colon (El-Ashmawy et al., 2018). A report suggested that licochalcone A (20, 40, 80 mg/kg) could reverse the increases in relative levels of inflammatory factors, including TNF-α, IL-1β and IL-6, MPO activity, and NO level and decrease GSH and SOD levels via the NF-κB signaling pathway and Nrf2 signaling pathway in DSS-induced colitis mice (Liu et al., 2018e). A report by Juan Zhang et al. suggested that licoflavone B (120 mg/kg) could repair the damage to the colonic barrier by inhibiting colonic cell apoptosis, protecting the expression of occludin, claudin-1, and ZO-1, and suppressing harmful bacteria (such as *Enterococcus*) and boosting beneficial microorganisms (such as *Bacteroides*). Furthermore, licoflavone B could suppress the expression of MAPK pathway-related proteins, including perk, p-p38, and pJNK(Zhang et al., 2022b). *In vitro* experiments have revealed that a flavonoid-rich extract of *Glycyrrhiza uralensis Fisch.* (6.25, 12.5, 25 mg/kg) could prevent and restore the intestinal barrier dysfunction induced by TNF-α in Caco-2 cell monolayers. Moreover, a flavonoid-rich extract of glycyrrhiza glabra could repair intestinal barrier damage by increasing the expression of occluding and ZO-1 protein in TNBS-induced colitis rats (Murugan et al., 2022). Chunying Huang et al. reported that polysaccharides from *Glycyrrhiza uralensis Fisch.* (100, 200, and 400 mg/kg) or the positive control drug sulfasalazine (200 mg/kg) could reduce intestinal permeability and inhibit the inflammatory response (IL-1, IL-6, TNF-α, IL-10 levels) in DSS-induced UC mice (Huang et al., 2022).

### 5.4 *Platycodon grandiflorum* (Jacq.) A. DC (Jie Geng)

*Platycodon grandiflorum* (Jacq.) A. DC is commonly used to relieve cough and asthma in clinical practice. The primary components of *Platycodon grandiflorum* (Jacq.) A. DC contain triterpenoid saponins, polysaccharides, and flavonoids, exhibiting antitussive, antiasthmatic, anti-inflammatory, antioxidant, and anti-tumor (Zuo et al., 2019). Platycodin D is a representative triterpenoid saponin component of *Platycodon grandiflorum* (Jacq.) A. DC. A study reported that platycodin D (10 mg/kg) was beneficial in DSS-induced colitis mice, which was related to macrophages. Further, platycodin D (2.5, 5 μM) could inhibit M1 macrophage polarization and promote M2 macrophage polarization in LPS-stimulated RAW 264.7 cells by PI3K/Akt, NF-κB, and AMPK-dependent signaling pathways (Guo et al., 2021). In a report by Yang liu et al., the MPO activity, contents of MDA, and expression of IL-1, IL-6, and TNF-α cytokines were inhibited significantly, and expression of IL-10 cytokine and levels of SOD were increased dramatically in UC mice treated with polysaccharides (100, 200, and 400 mg/kg) from *Platycodon grandiflorum* (Jacq.) A. DC (Liu et al., 2022b). In addition, *Platycodon grandiflorum* (Jacq) A. DC root fermentation broth (0.5, 1 ml per head per day) was suggested to improve UC prognosis by regulating the AMPK/NF-κB/NLRP3 signaling pathway (Wang et al., 2022).

### 5.5 *Panax ginseng* C.A.Mey. (Ren Shen)

*Panax ginseng* C.A.Mey. is a Chinese drug with a high medicinal value. It has the effects of invigorating vital energy, strengthening the spleen, tonifying the lung, engendering liquid, allaying thirst, AND tranquilizing the mind. Modern studies showed that *Panax ginseng* C.A.Mey. has various pharmacological effects such as anti-aging, antioxidant, anti-tumor, and immune enhancement (Yu et al., 2019a). Ginsenosides are the main activity components of *Panax ginseng* C.A.Mey., exhibiting immunomodulatory and anti-inflammatory activity against UC(Shi, 2011). A study reported that ginsenoside Rd (10, 20 and 40 mg/kg) could improve the syndrome of TNBS-induced colitis rats by enhancing the oxidation resistance of injured colons and inhibiting neutrophil infiltration (Yang et al., 2012). Meanwhile, ginsenoside Rd (10, 20 and 40 mg/kg) could inhibit the NLRP3 inflammasome through the AMPK/ULK1-autophagy signaling pathway in DSS-induced colitis mice (Liu et al., 2018f). In addition, ginsenoside Rd (20 mg/kg) could reduce the levels of TNF-α, IFN-γ, IL-6, IL-12/23p40, IL-17 A, and the expression of relative proteins including pJNK, p-p38, pIκBα, and p65 of NF-κB and p38 MAPK pathways, which eventually improve the condition of UC mice (Qu et al., 2022b). Weiwei hao et al. reported that ginsenoside Rg1 (50 and 200 mg/kg) could improve the hypercoagulability and microcirculation in DSS-induced colitis mice (Hao et al., 2013). Further, ginsenoside Rg1 (200 mg/kg) could ameliorate the symptoms in DSS-induced UC mice by regulating M1/M2 macrophage polarization associated with inhibition of the Nogo-B/RhoA signaling pathway, microbiota composition, and the balance of Treg/Th9 cells (Long et al., 2022a; Long et al., 2022b). Ginsenoside Rh2 (20 mg/kg) exhibited a potential therapeutic effect on UC, decreasing the mRNA expression of IL-6, TNFa, and INFc in the DSS-treated colon, and augmenting the TGFb signaling pathway (Ye et al., 2014b). Xuanqing Chen and colleagues suggested that ginsenoside Rh2 (50 mg/kg) is potentially valuable for treating UC, and its mechanism involves the downregulation of STAT3/miR-214 levels (Chen et al., 2021). Meanwhile, Yu Xu et al. successfully encapsulated ginsenoside Rh2 to form Rh2 nanoparticles that exhibit strong anti-inflammatory activity via significantly inhibiting the overproduction of nitric oxide (NO) and inflammatory cytokines (TNF-α, IL-1β and IL-6). Further, the Rh2 nanoparticles could regulate the oxidant stress levels and intestinal flora of UC mice (Xu et al., 2022). Zhiwei miu et al. reported that ginsenoside Rg3 (40 mg/kg) has significant therapeutic effects on DSS-induced colitis mice. It could regulate the imbalance of Th1/Th2 by decreasing TNF-α and IL-6 levels, increasing IL-10 levels, and suppressing the NF-κB signaling pathway by decreasing the expression of p-NF-κB p65 and NF-κB p65 (Miao et al., 2019). Evelyn Saba and colleagues found that ginsenoside Rg3 (20 mg/kg) could decrease the expression of pro-inflammatory mediators and cytokines including NO, IL-1β, IL-5, IL-13, and TNF-α, and levels of NLRP3 inflammasome in DSS-induced colitis mice (Saba et al., 2020). Mi Tian et al. reported that ginsenoside RK3 (20, 40 and 60 mg/kg) protected intestinal barrier function and inhibited NLRP3 inflammasome expression in DSS-induced colitis mice by regulating the MPO and iNOS activities and expression of TNF-α, IL-1β, IL-6, NLRP3, ASC, and Caspase-1 (Tian et al., 2020).

### 5.6 *Amomum villosum* Lour. (Sha Ren)

*Amomum villosum* Lour. mainly contains volatile oil, flavonoids, and phenolic acid, exhibiting various pharmacology activities, including gastrointestinal protection, antioxidant, antibacterial, and blood pressure-lowering effects (Qu et al., 2021). Among these, volatile oil and flavonoids from *Amomum villosum* Lour. are the potential drugs used to treat peptic colitis (Qu et al., 2021). Studies reported that volatile oil (0.84, 1.6 g/kg) from *Amomum villosum* Lour. could alleviate the oxidative damage caused by increasing SOD activity and levels of GSH-Px, decreasing the levels of NOS and expression of iNOS, decreasing colonic cell-to-cell adhesion by inhibiting the expression of ICAM, and suppressing inflammatory response by decreasing the expression of TNF-α and NF-κB p65 (Zhao, 2009; Zhu et al., 2009).

### 5.7 *Dioscorea opposita* Thunb. (Shan Yao)

*Dioscorea opposita* Thunb. can help tonify spleen and stomach, benefit the lung and generating fluid, tonify the kidney and essence. Modern studies showed that the chemical composition of yam majorly included polysaccharides, amino acid, fatty acid, dioscin, and polyphenols (Chen et al., 2020). A study showed that polyphenols (240 mg/kg) from *Dioscorea opposita* Thunb. could protect against the DSS-induced colitis mice. Administering *Dioscorea opposita* Thunb. before modeling markedly mitigated colitis as well as intestinal mucosal damage and apoptosis of colonic epithelial cells by regulating the expression of occludin, caspase-8, and COX-2 (Li et al., 2021c).

### 5.8 *Coix lacryma-jobi* L.var. *mayuen* (Roman.) Stapf (Yi Yi Ren)

*Coix lacryma-jobi* L. var. *mayuen* (Roman.) Stapf, as a TCM with a homology of medicine and food, has anti-inflammatory, anti-obesity, anti-tumor, and antiallergic activity. It primarily contains lipid acid, polysaccharides, lignans, and phenols (Li et al., 2020c). A study showed that the extract (1.5 g/kg) of *Coix lacryma-jobi* L. var. *mayuen* (Roman.) Stapf has a protective effect on DSS-induced UC rats, which may be related to the antioxidant potential (Hao et al., 2012). Further, Qilyu Zhou and colleagues found that the feed of *Coix lacryma-jobi* L. var. *mayuen* (Roman.) Stapf could not only relieve inflammatory cytokine secretion and alleviate oxidative stress but also change the innate immune cell proportion, which eventually ameliorated immune function disorders for treating UC mice (Zhou et al., 2021a).

### 5.9 Metabolites of herbs in SLBZP

Therapeutic effects of many constituents of herbs may depend on the transformative components after metabolism *in vivo* rather than prototype components (Yu et al., 2019b). In recent years, in addition to prototype components, metabolites of herbs can often be one of the substances that contribute to the efficacy of the herbs or prescriptions (Kang et al., 2020). Nowadays, lots metabolites of Chinese herbal medicines, such as ginsenoside compound K (CK), have shown various beneficial therapy effects on UC. Ginsenoside CK is the main metabolite of the protopanaxadiol type of ginsenoside (Liu et al., 2022c). Juan Li et al. and colleagues found that Ginsenoside CK (5, 10 and 20 mg/kg) could promote the recovery of the progression of UC and inhibit the inflammatory responses by suppressing NF-kB activation or regulating the activation of macrophages (Li et al., 2014b). Meanwhile, studies have found that the gut microbial metabolite CK had significant anti-inflammatory effects on UC even at low concentrations, compared to its parent ginsenoside Rb1 (Wang et al., 2018a). In addition, Hao Cai et al. found that the metabolites (Structures were shown in Figure 7) of *Atractylodes macrocephala* Koidz. including atractylenolide I-M1, atractylenolide I-M2, atractylenolide III-M1, atractylenolide III-M2, atractylenolide III-M3 and atractylenolide II-M1 were high degree correlated with the levels of TNF-α, IL-6, IL-10, and TGF-β1, demonstrating strong anti-UC effects (Cai et al., 2019). And further study is required to verify the effectiveness of Metabolites of *Atractylodes macrocephala* Koidz. for treating UC. Nevertheless, no data on the metabolites of SLBZP are currently available. Prototype components and metabolites of SLBZP that contribute to the therapeutic effects of UC and the mechanism need to be further investigated.

## 6 Summary and outlook

As one of the classic prescriptions for strengthening the spleen and clearing dampness, SLBZP has beneficial effects on the prevention and treatment of UC. Numerous studies have demonstrated that SLBZP alleviates the symptoms and decreases the recurrence rate of UC, thereby improving the quality of life. The mechanism of action of SLBZP could be attributed to anti-inflammatory, antioxidant, and immunomodulatory effects, as well as repair of intestinal mucosal damage and protection of gut mucosal barrier, promotion of BMSCs migration to the colonic mucosa, regulation of some signal pathways, and regulation of the balance of gut microbiota. These studies not only provide a theoretical basis for the clinical application of SLBZP in the treatment of UC and further research on UC mechanisms but also provide more choices for the prevention and treatment of UC, improving the possibility of curing UC. However, these studies still have some limitations and ambiguities. According to the current research, eight herbs and their ingredients have been reported to exert therapeutic effects, suggesting a multi-component, multi-target, and multi-pathway mode of action of SLBZP in treating UC (As shown in Figure 6).

However, the ingredients that contribute to the therapeutic effects and the mechanism underlying their synergistic activity remain unclear. Moreover, only characterized components (such as ginsenoside Rg1, Rb1, Re, and atractylenolide III) and HPLC fingerprint of SLBZP were analyzed and reported in some studies (Liu and Zhu 2018; Wang et al., 2018b), and the overall components of SLBZP and its pharmacological effects and mechanism remains to be further investigated. Additionally, since the mechanism of SLBZP has not yet been elucidated, further large-scale evaluations assessing the efficacy and safety of SLBZP and its combination with other drugs to prevent and treat UC are required. Furthermore, the ideal dosage form and treatment duration of SLBZP was inconsistent in the clinic. There may be a difference in the quality of Chinese drugs, which could affect the accuracy of our findings. Furthermore, several animal models that have been developed accurately represent certain aspects of UC. However, they do not completely mimic the human UC pathology, especially the TCM syndromes of UC, which can affect our evaluation of the therapeutic effects of SLBZP in the treatment of UC.

In a sum, SLBZP has shown a broad prospect in the prevention and treatment of UC, and further research is required in the future. That should mainly focus on the following aspects: 1) Large sample prospective cohort studies are performed to clarify the clinical efficacy and safety of SLBZP and combination with other drugs in treatment of UC; 2) Researchers should strengthen the study of molecular biological mechanism of active ingredients and its synergistic actions, clarifying the mechanism of SLBZP in treatment of UC by multi-component, multi-target and multi-pathway.
