# Supplementary material for: Traditional Chinese medicine prescription Shenling BaiZhu powder to treat ulcerative colitis: Clinical evidence and potential mechanisms
Source: Front Pharmacol. 2022 Sep 6;13:978558. doi: 10.3389/fphar.2022.978558 (PMC9494158; doi:10.3389/fphar.2022.978558)
Supplement: Supplementary file 1 [file Table1.DOCX]

**Table 1 The relative cytokines in UC were regulated by SLBZP**

| Cytokines | Roles in UC | Sources | Regulation of SLBZP |
| --- | --- | --- | --- |
| TNF-α | Pro-inflammatory, innate immunity | Macrophages | Down-regulated |
| IL-3 | Induce differentiation of various pluripotent and multipotent stem and progenitor cells | T cells, Natural killer T cells | Down-regulated |
| IL-6 | Pro-inflammatory, innate immunity | Macrophages | Down-regulated |
| IL-8 | Neutrophil chemo-attractant | T cells, fibroblasts, endothelial cells, macrophages | Up-regulated |
| IL-10 | Anti-inflammatory | T cells, B cells | Up-regulated |
| IL-13 | Pro-inflammatory, disruption of epithelial barrier | Natural killer T cells | Down-regulated |
| IL-33 | Induction of Th2 cytokines | Epithelial cells | Down-regulated |
| TGF-β | Anti-inflammatory, activation of Th2 cells | Mononuclear cells | Up-regulated |
| IL-1β | Pro-inflammatory | Macrophages | Down-regulated |
| IL-4 | Anti-inflammatory | Th2 cells | Up-regulated |
| IL -17 | Pro-inflammatory | Th17, monocytes, neutrophils, T CD8+, NK cells | Down-regulated |
| IL-23 | Pro-inflammatory | Macrophages, dendritic cells | Down-regulated |
