# Supplementary material for: Traditional Chinese medicine prescription Shenling BaiZhu powder to treat ulcerative colitis: Clinical evidence and potential mechanisms
Source: Front Pharmacol. 2022 Sep 6;13:978558. doi: 10.3389/fphar.2022.978558 (PMC9494158; doi:10.3389/fphar.2022.978558)
Supplement: Supplementary file 2 [file Table2.DOCX]

**Table 2 The composition and main components of SLBZP**

| Herb (Local Name) | Medicinal parts | Main chemical components |
| --- | --- | --- |
| *Panax ginseng* C. A. Mey.  (Ren Shen) | Roots and rhizomes | Triterpenoids, polysaccharides, volatile, organic acid |
| *Atractylodes macrocephala* Koidz. (Bai Zhu) | Rhizomes | Triterpenoids, sesquiterpenoids, polyacetylenes, polysaccharides |
| *Poria cocos* (SchW.) Wolf. (Fu Ling) | Sclerotium | Triterpenoids, polysaccharides, sterols, volatile |
| *Glycyrrhiza uralensis* Fisch. or *Glycyrrhiza inflata* Bat. or Glycyrrhiza glabra L.  (Gan Cao) | Roots and rhizomes | Triterpenoids, polysaccharides, flavonoids, coumarins |
| *Platycodon grandiflorum* (Jacq.) A. DC.  (Jie Geng) | Roots | Triterpenoid saponins, flavonoids |
| *Coix lacryma-jobi* L. var. mayuen (Roman.) Stapf.  (Yi Yi Ren) | Seed kernels | Fatty acid and esters, polysaccharides, flavonoids |
| *Nelumho nucifera* Gaertn. (Lian Zi) | Seeds | Alkaloid, flavonoids |
| *Dioscorea opposita* Thunb. (Shan Yao) | Rhizomes | Polysaccharides, amino acid, fatty acid |
| *Amomum villosum* Lour. or *Amomum villosum* Lour. var. *xanthioides* T. L. Wu et Senjen or *Amomum longili*gulare T. L. Wu (Sha Ren) | Fruits | Volatile, flavonoids, phenolic acid |
| *Dolichos lablab* L. (Bai Bian Dou) | Seeds | Polysaccharides, lipid, protein |
